# Supplementary material for: Motivational Interviewing: A High-Yield Interactive Session for Medical Trainees and Professionals to Help Tobacco Users Quit
Source: MedEdPORTAL. 2019 Aug 23;15:10831. doi: 10.15766/mep_2374-8265.10831 (PMC6868517; doi:10.15766/mep_2374-8265.10831)
Supplement: Supplementary file 1 — A. MI Presentation.pptx B. MI Workshop Scenarios.docx C. Checklist for MI.docx D. MI Laminated Card.pptx E. Resident Survey.docx F. MI Facilitator Guide.docx [file mep-15-10831-s001.zip › A._MI_Presentation.pptx]

## Slide 1
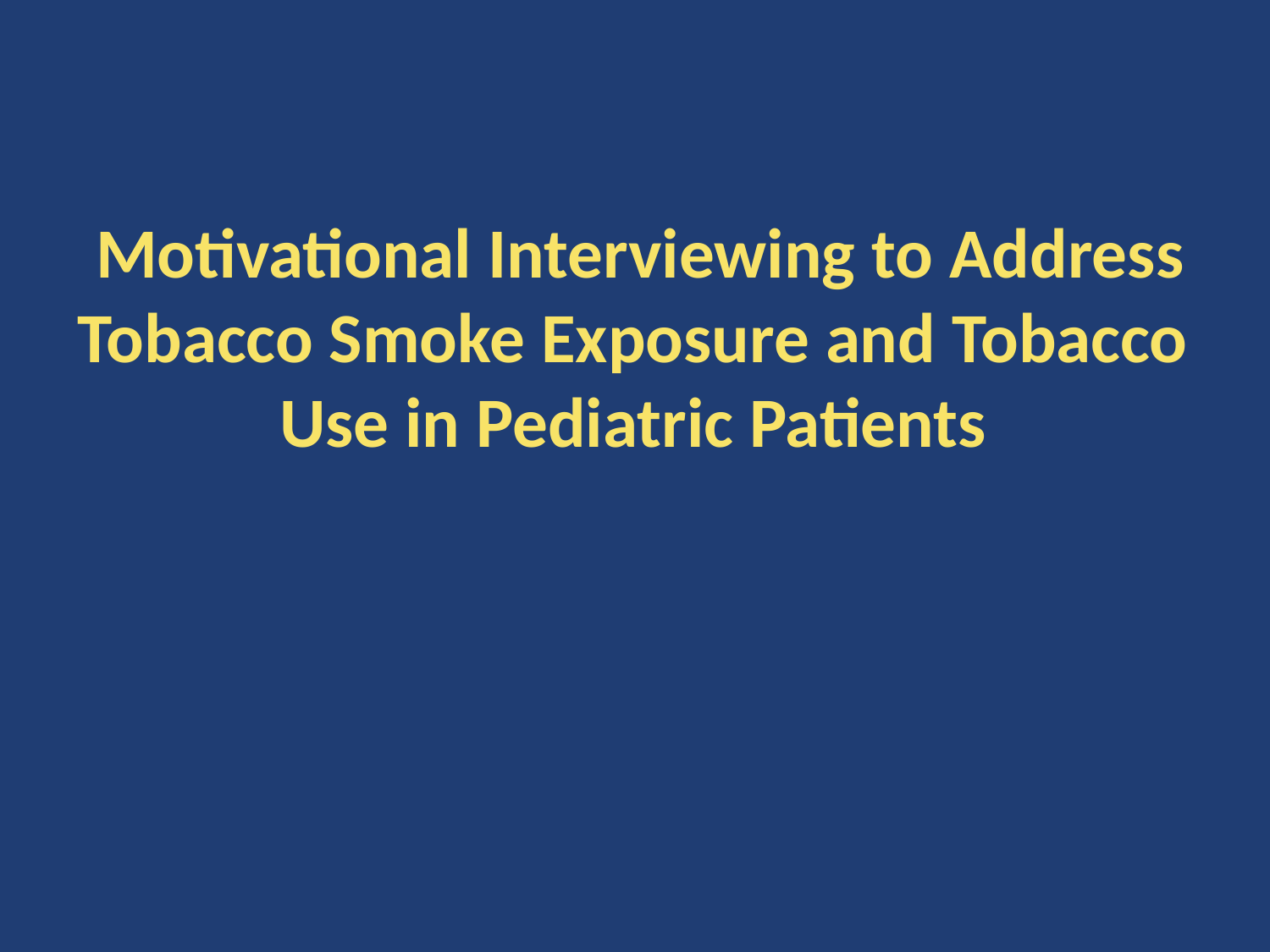

# Motivational Interviewing to Address Tobacco Smoke Exposure and Tobacco Use in Pediatric Patients

## Slide 2
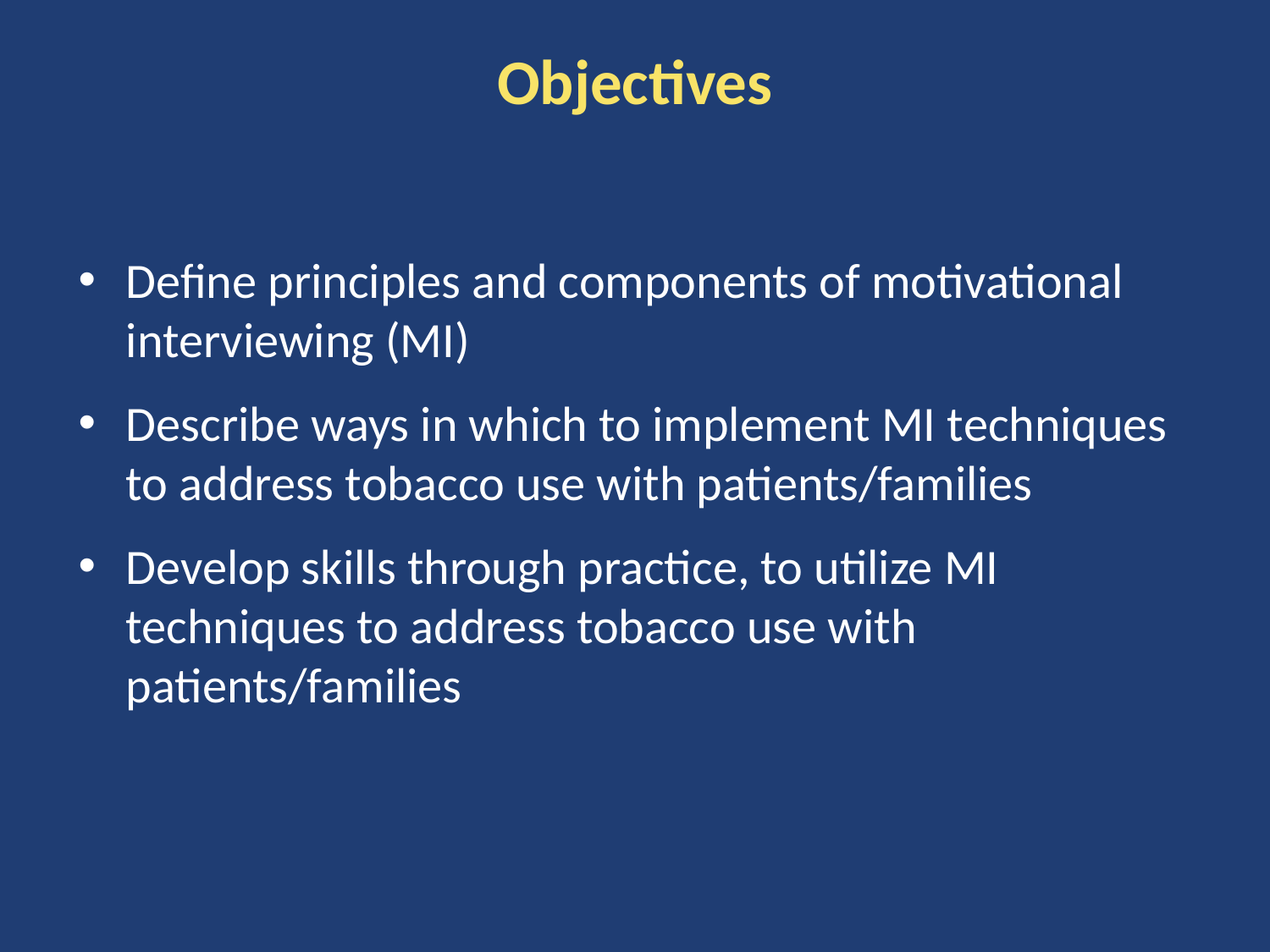

# Objectives
Define principles and components of motivational interviewing (MI)
Describe ways in which to implement MI techniques to address tobacco use with patients/families
Develop skills through practice, to utilize MI techniques to address tobacco use with patients/families

## Slide 3
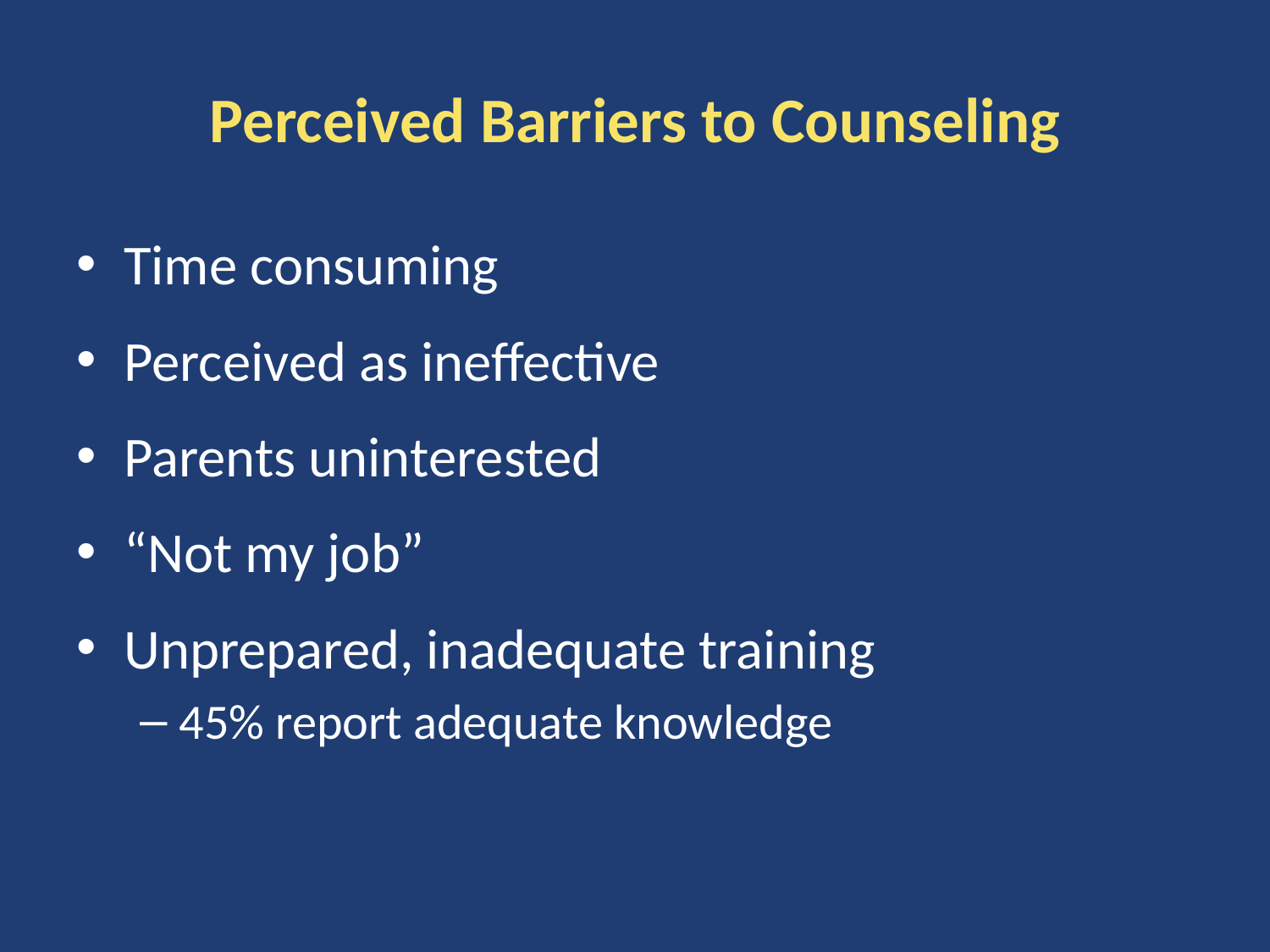

# Perceived Barriers to Counseling
Time consuming
Perceived as ineffective
Parents uninterested
“Not my job”
Unprepared, inadequate training
45% report adequate knowledge

## Slide 4
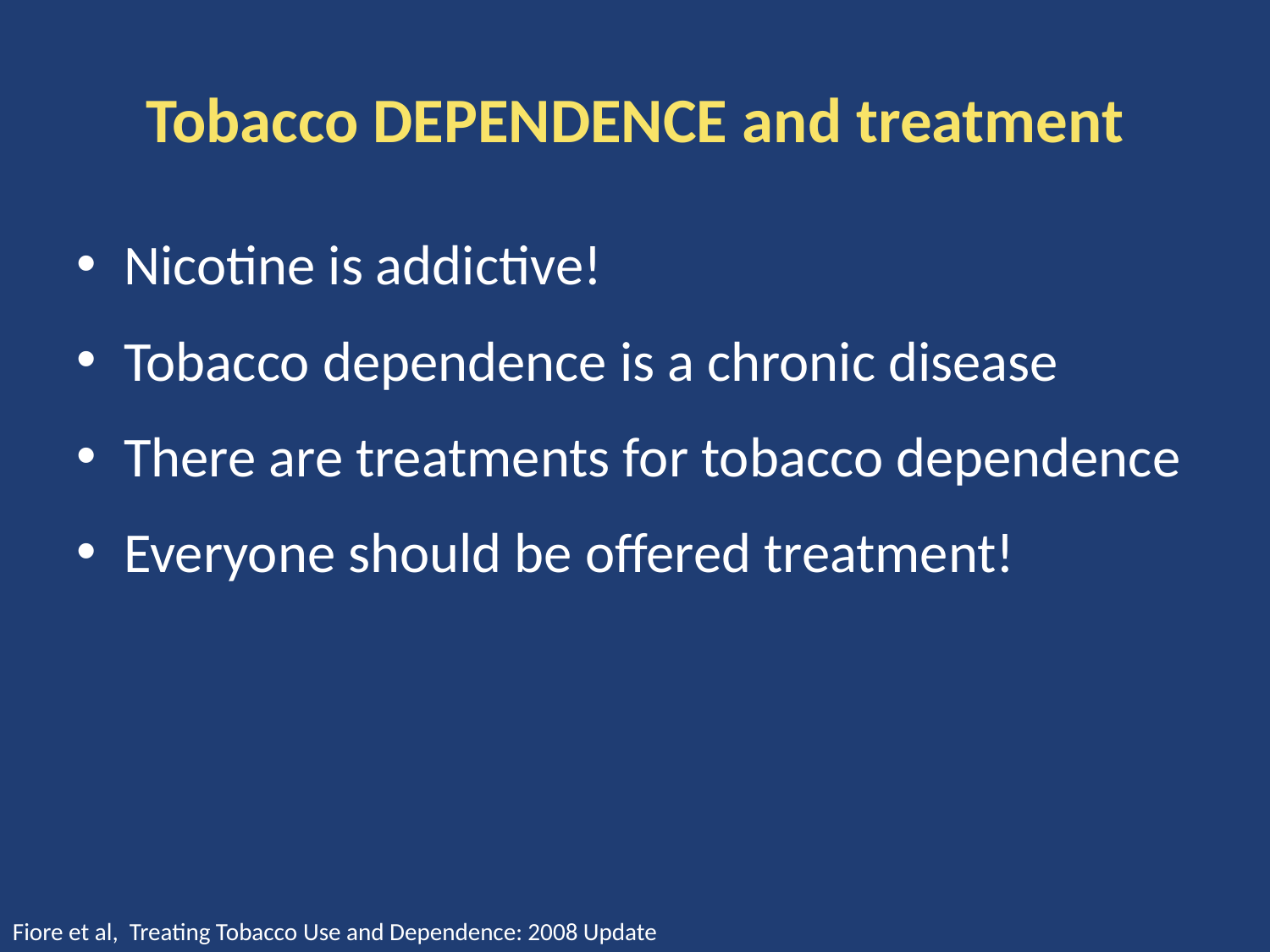

# Tobacco DEPENDENCE and treatment
Nicotine is addictive!
Tobacco dependence is a chronic disease
There are treatments for tobacco dependence
Everyone should be offered treatment!
Fiore et al, Treating Tobacco Use and Dependence: 2008 Update

## Slide 5
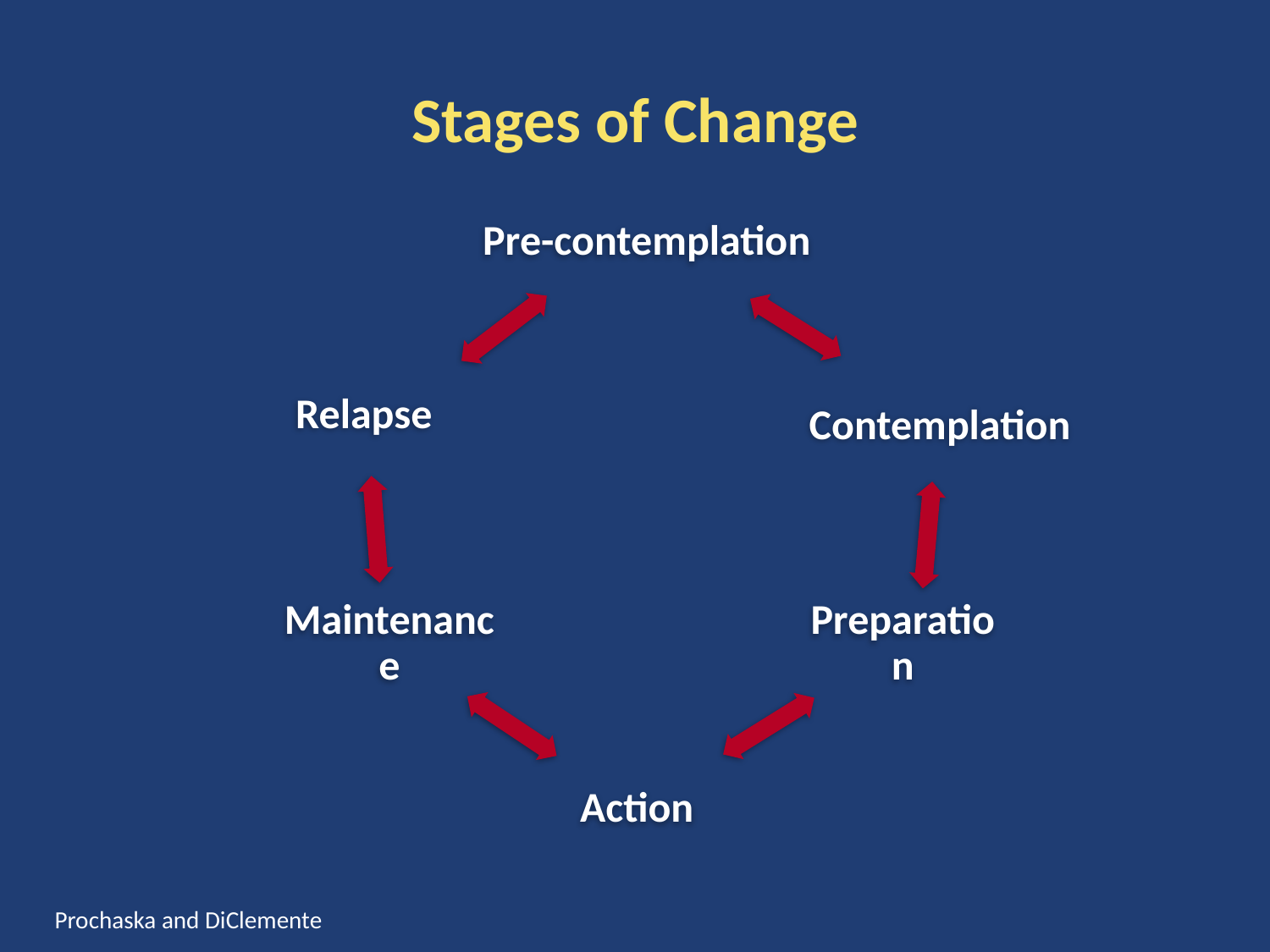

# Stages of Change
Prochaska and DiClemente

## Slide 6
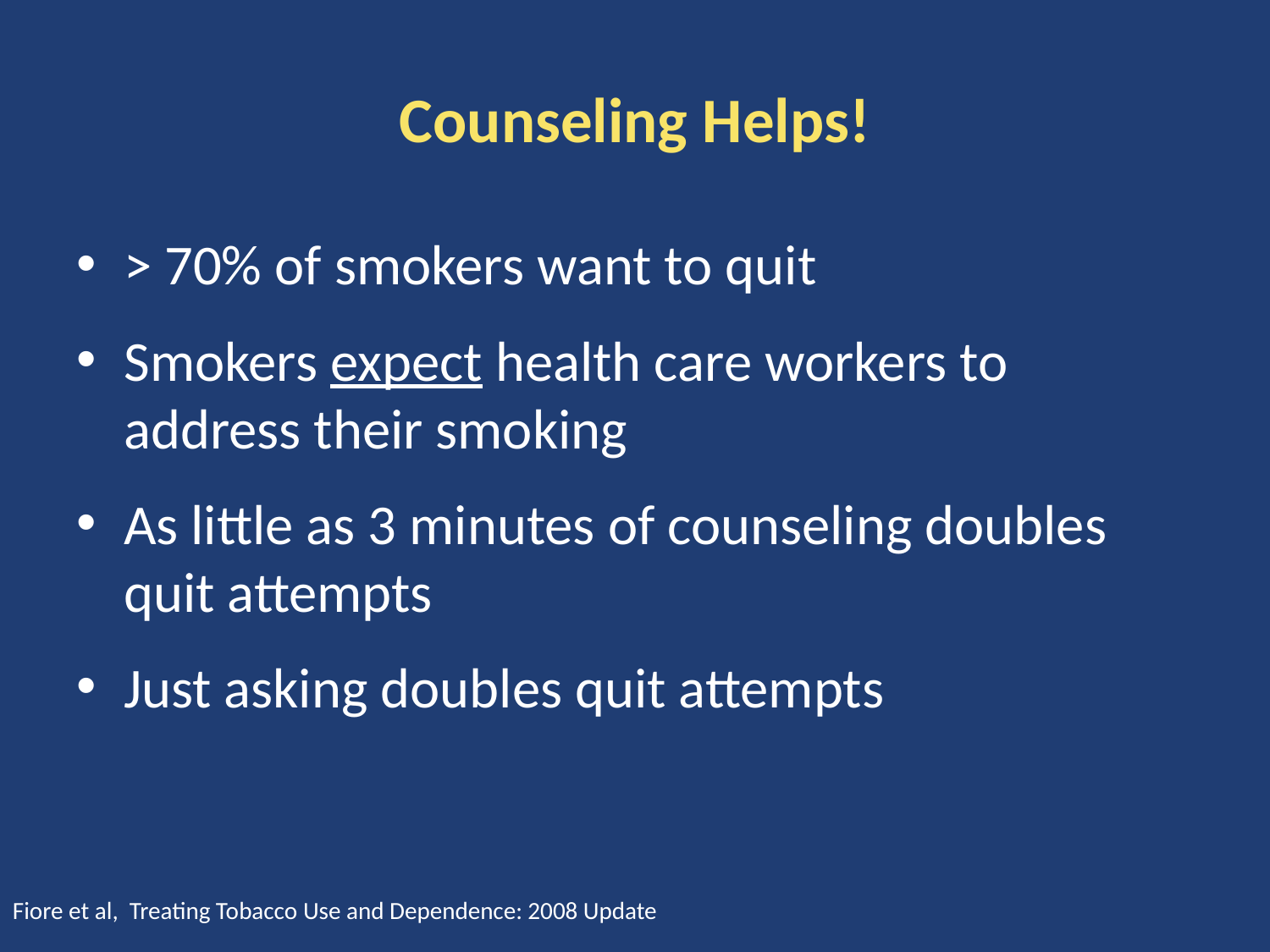

# Counseling Helps!
> 70% of smokers want to quit
Smokers expect health care workers to address their smoking
As little as 3 minutes of counseling doubles quit attempts
Just asking doubles quit attempts
Fiore et al, Treating Tobacco Use and Dependence: 2008 Update

## Slide 7
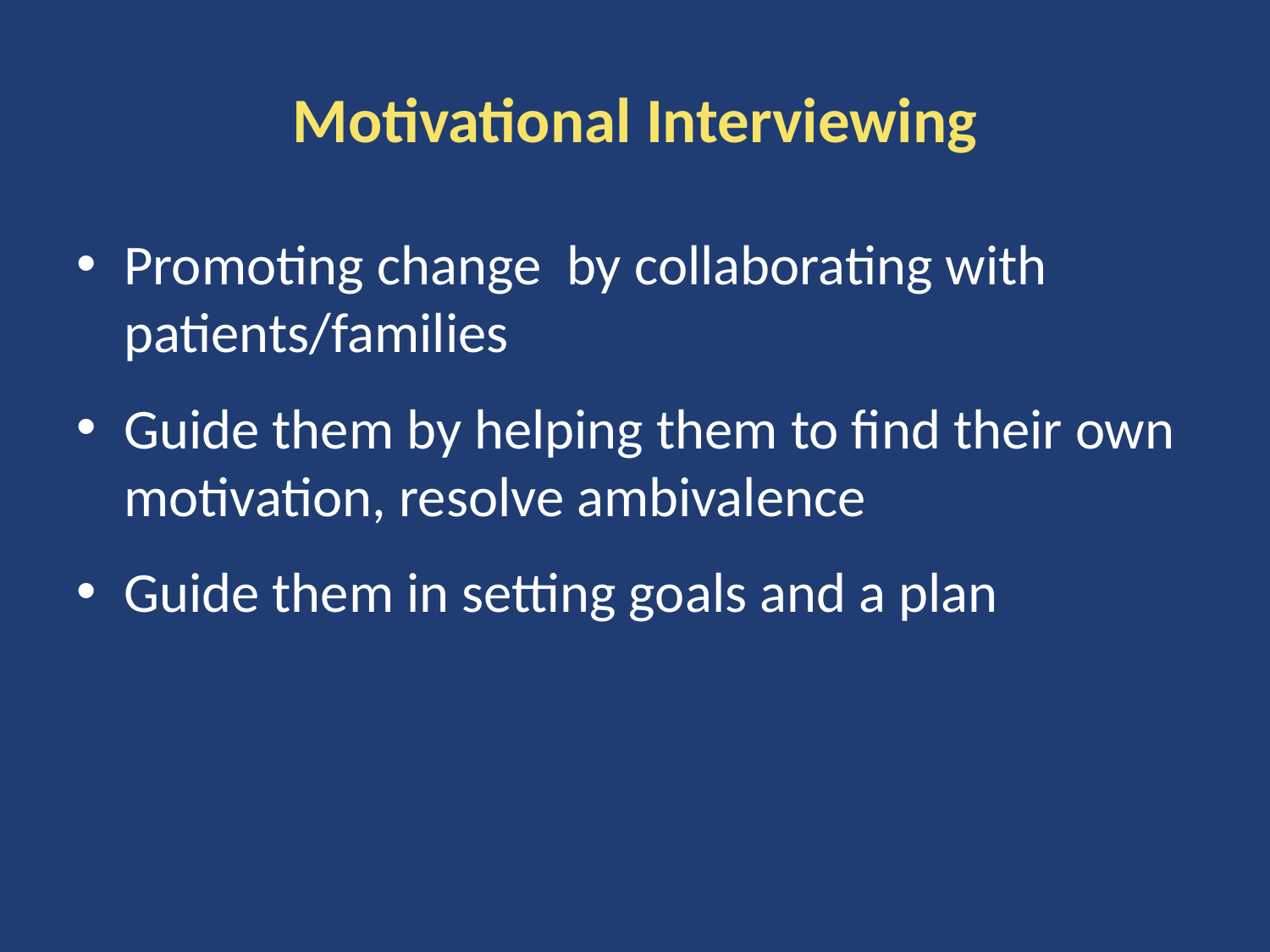

# Motivational Interviewing
Promoting change by collaborating with patients/families
Guide them by helping them to find their own motivation, resolve ambivalence
Guide them in setting goals and a plan

## Slide 8
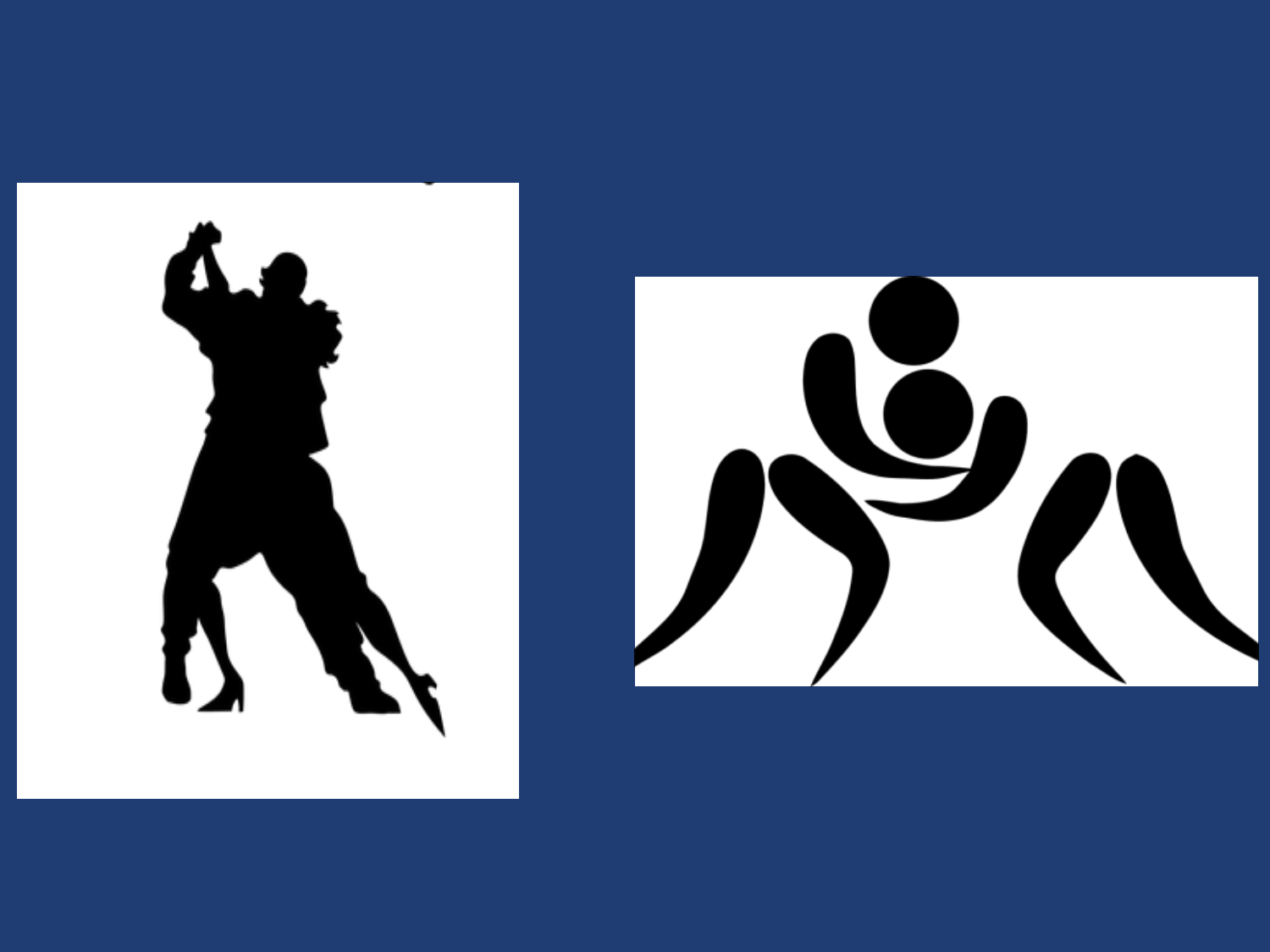

## Slide 9
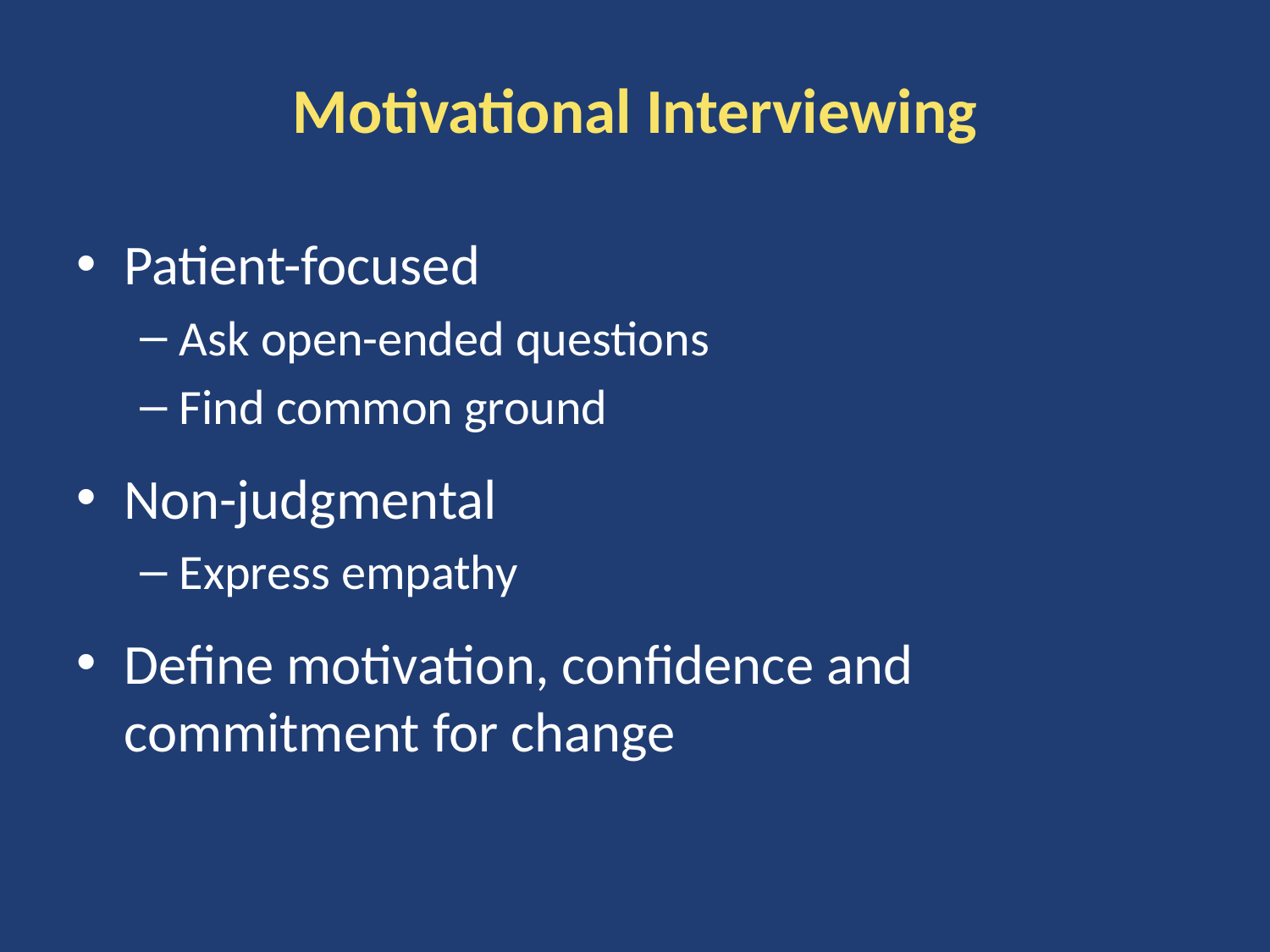

# Motivational Interviewing
Patient-focused
Ask open-ended questions
Find common ground
Non-judgmental
Express empathy
Define motivation, confidence and commitment for change

## Slide 10
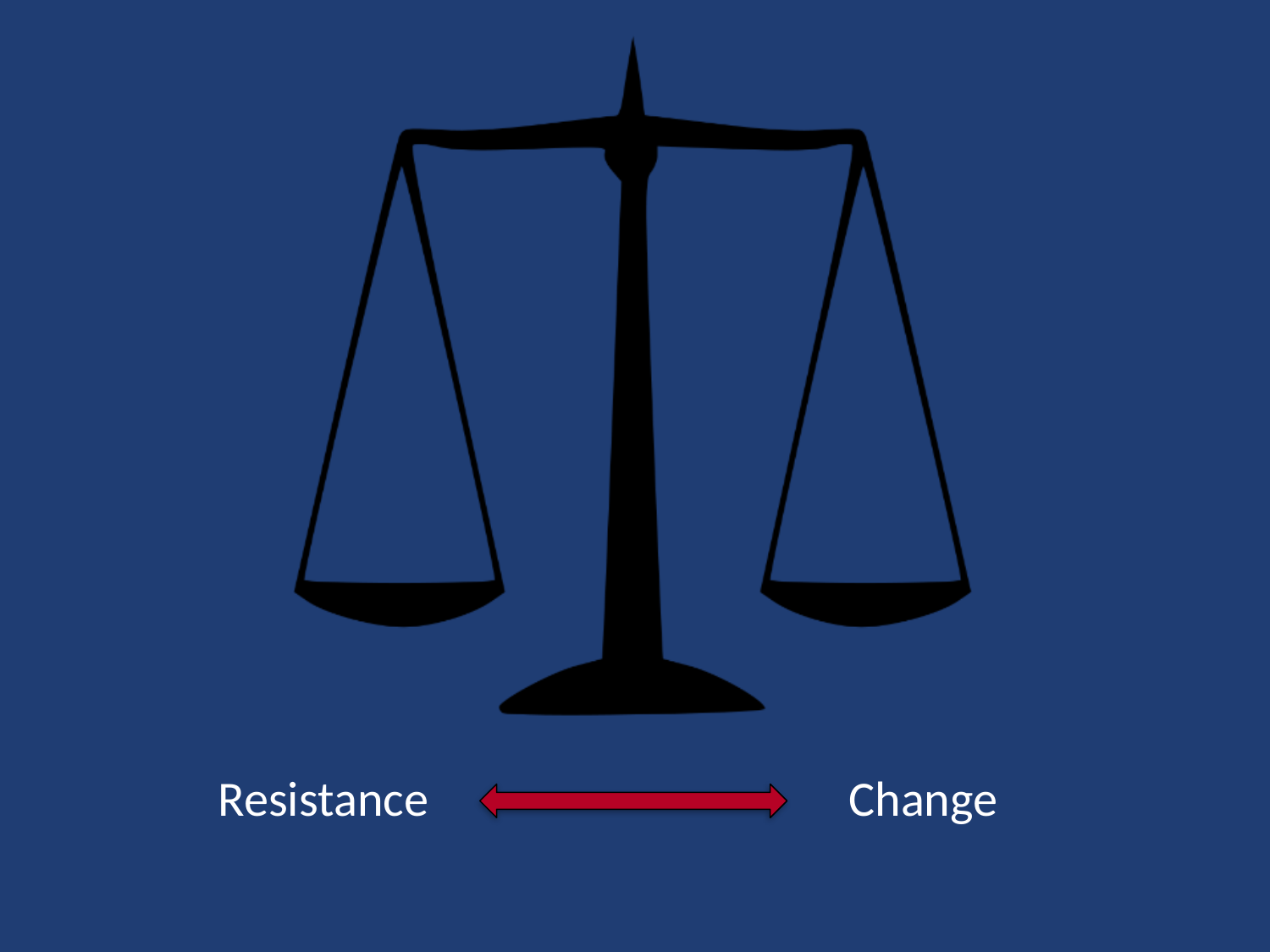

Resistance
Change

## Slide 11
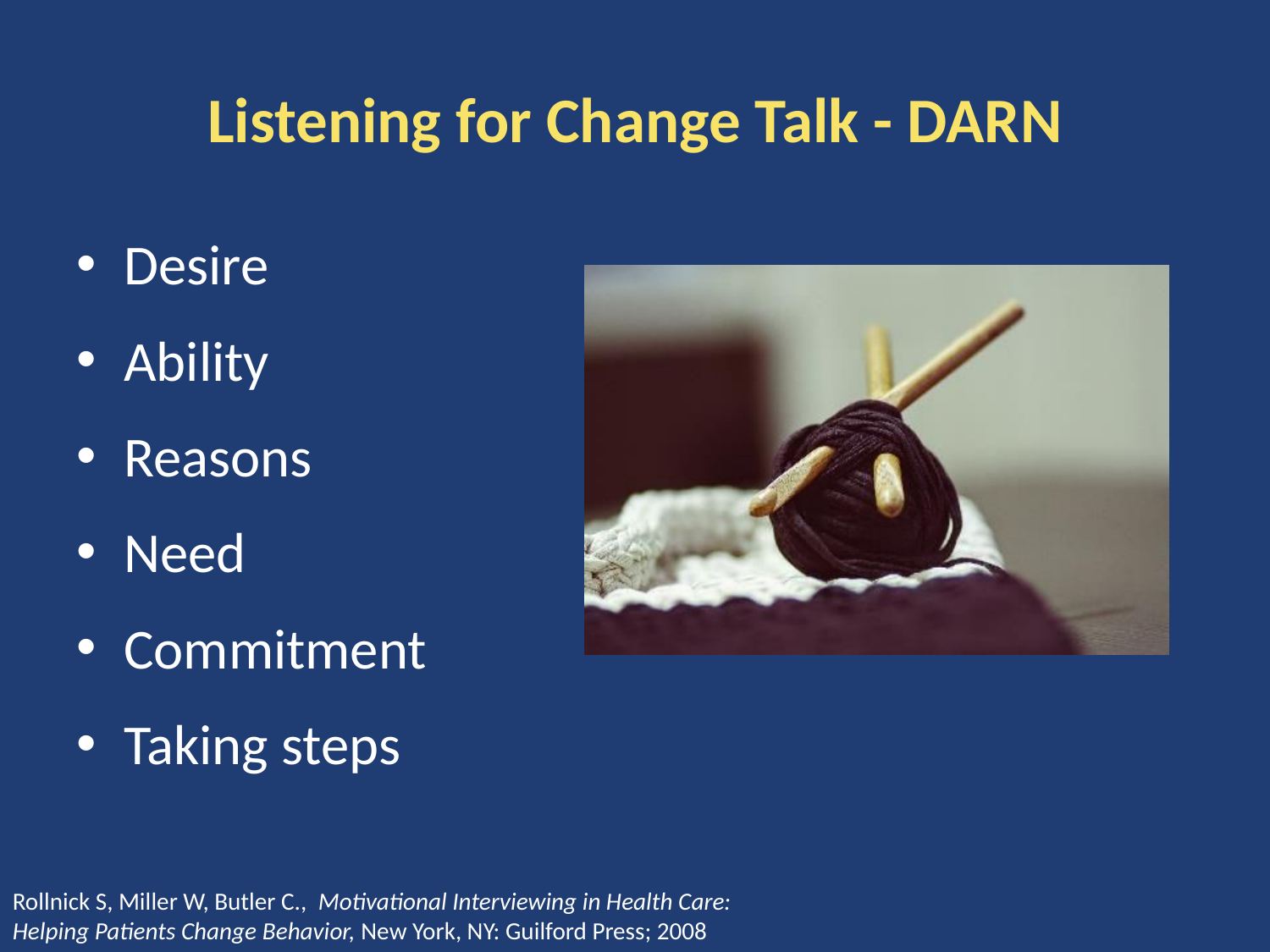

# Listening for Change Talk - DARN
Desire
Ability
Reasons
Need
Commitment
Taking steps
Rollnick S, Miller W, Butler C., Motivational Interviewing in Health Care:
Helping Patients Change Behavior, New York, NY: Guilford Press; 2008

## Slide 12
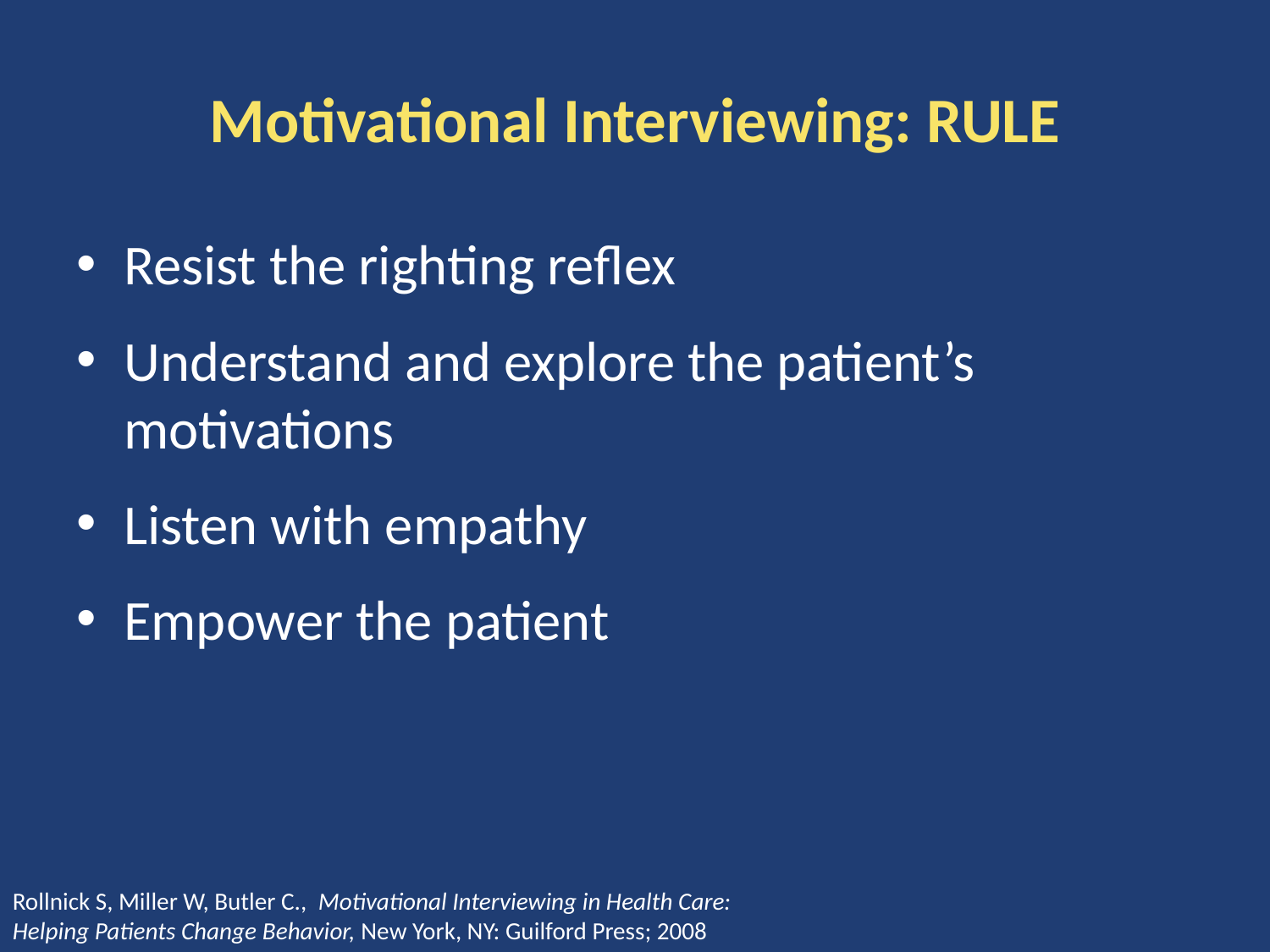

# Motivational Interviewing: RULE
Resist the righting reflex
Understand and explore the patient’s motivations
Listen with empathy
Empower the patient
Rollnick S, Miller W, Butler C., Motivational Interviewing in Health Care:
Helping Patients Change Behavior, New York, NY: Guilford Press; 2008

## Slide 13
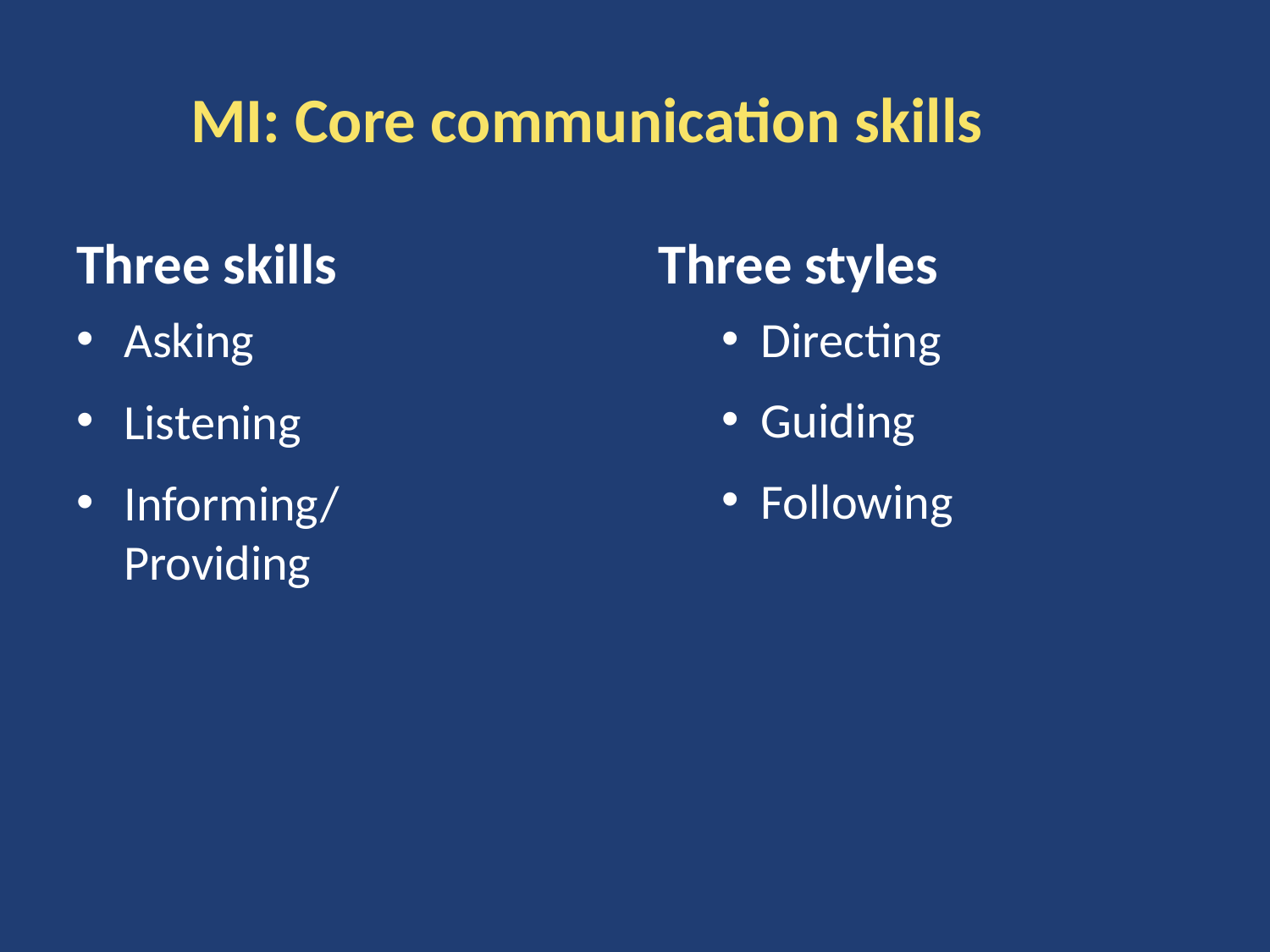

# MI: Core communication skills
Three skills
Three styles
Asking
Listening
Informing/Providing
Directing
Guiding
Following

## Slide 14
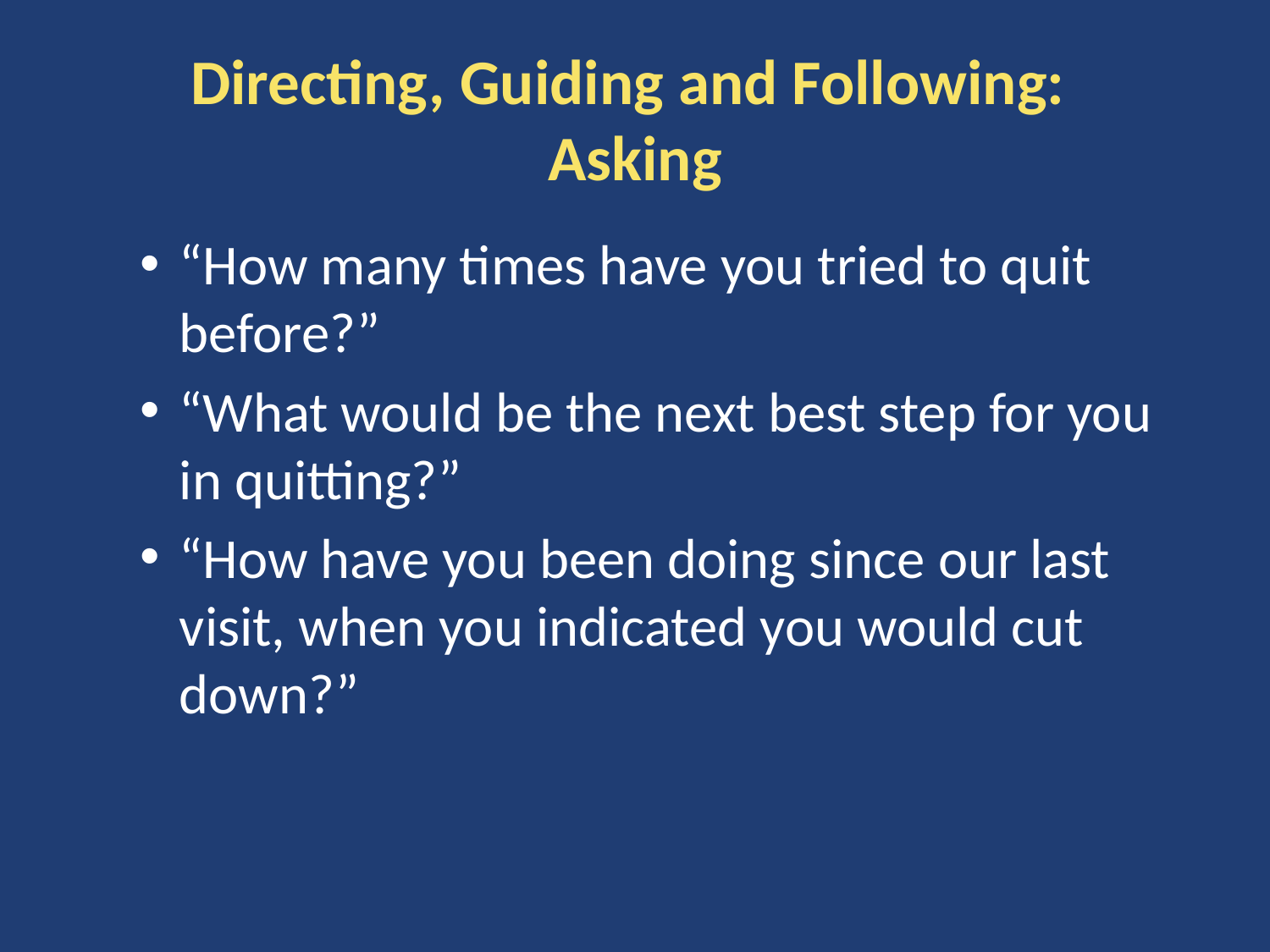

# Directing, Guiding and Following: Asking
“How many times have you tried to quit before?”
“What would be the next best step for you in quitting?”
“How have you been doing since our last visit, when you indicated you would cut down?”

## Slide 15
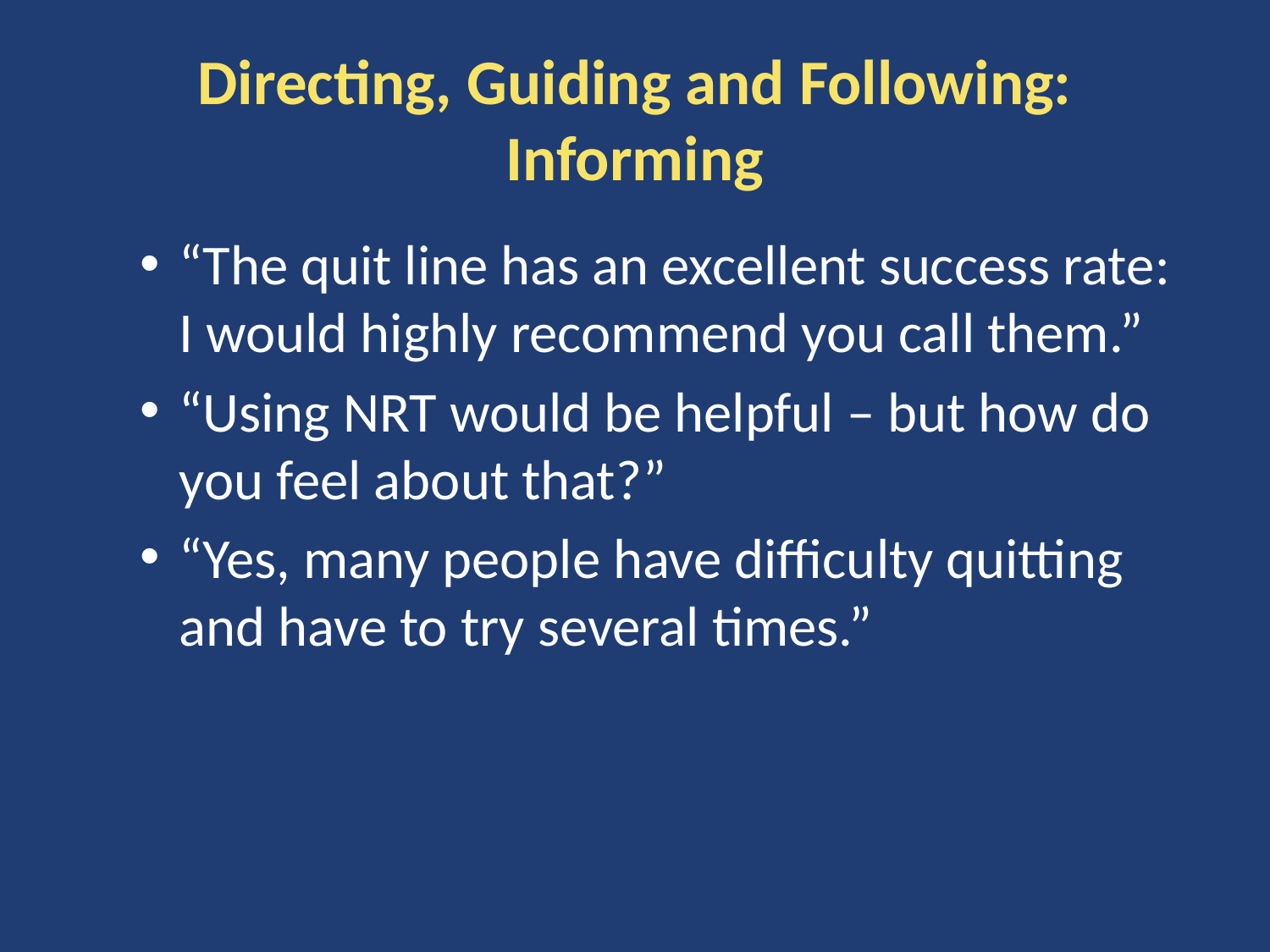

# Directing, Guiding and Following: Informing
“The quit line has an excellent success rate: I would highly recommend you call them.”
“Using NRT would be helpful – but how do you feel about that?”
“Yes, many people have difficulty quitting and have to try several times.”

## Slide 16
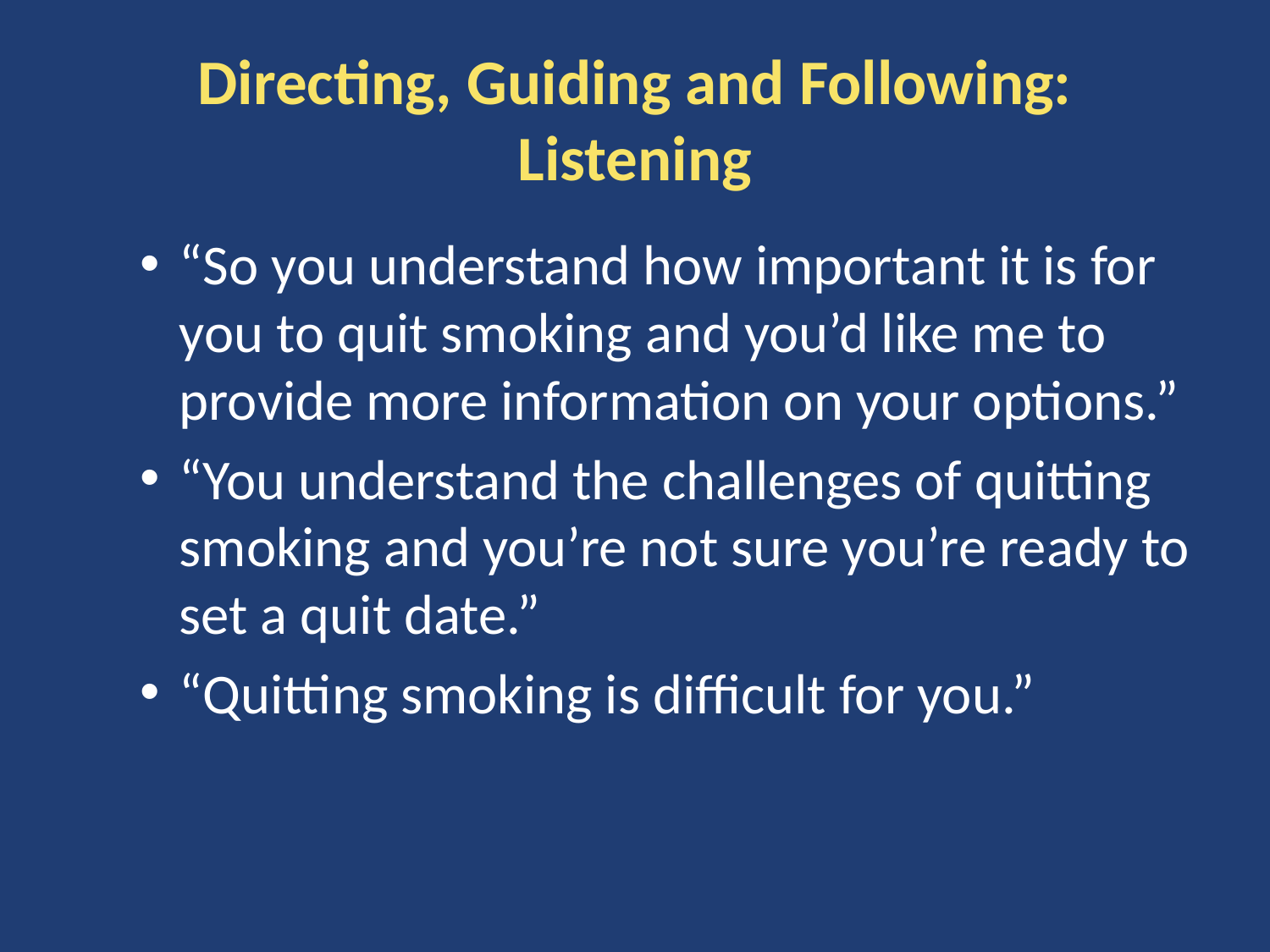

# Directing, Guiding and Following: Listening
“So you understand how important it is for you to quit smoking and you’d like me to provide more information on your options.”
“You understand the challenges of quitting smoking and you’re not sure you’re ready to set a quit date.”
“Quitting smoking is difficult for you.”

## Slide 17
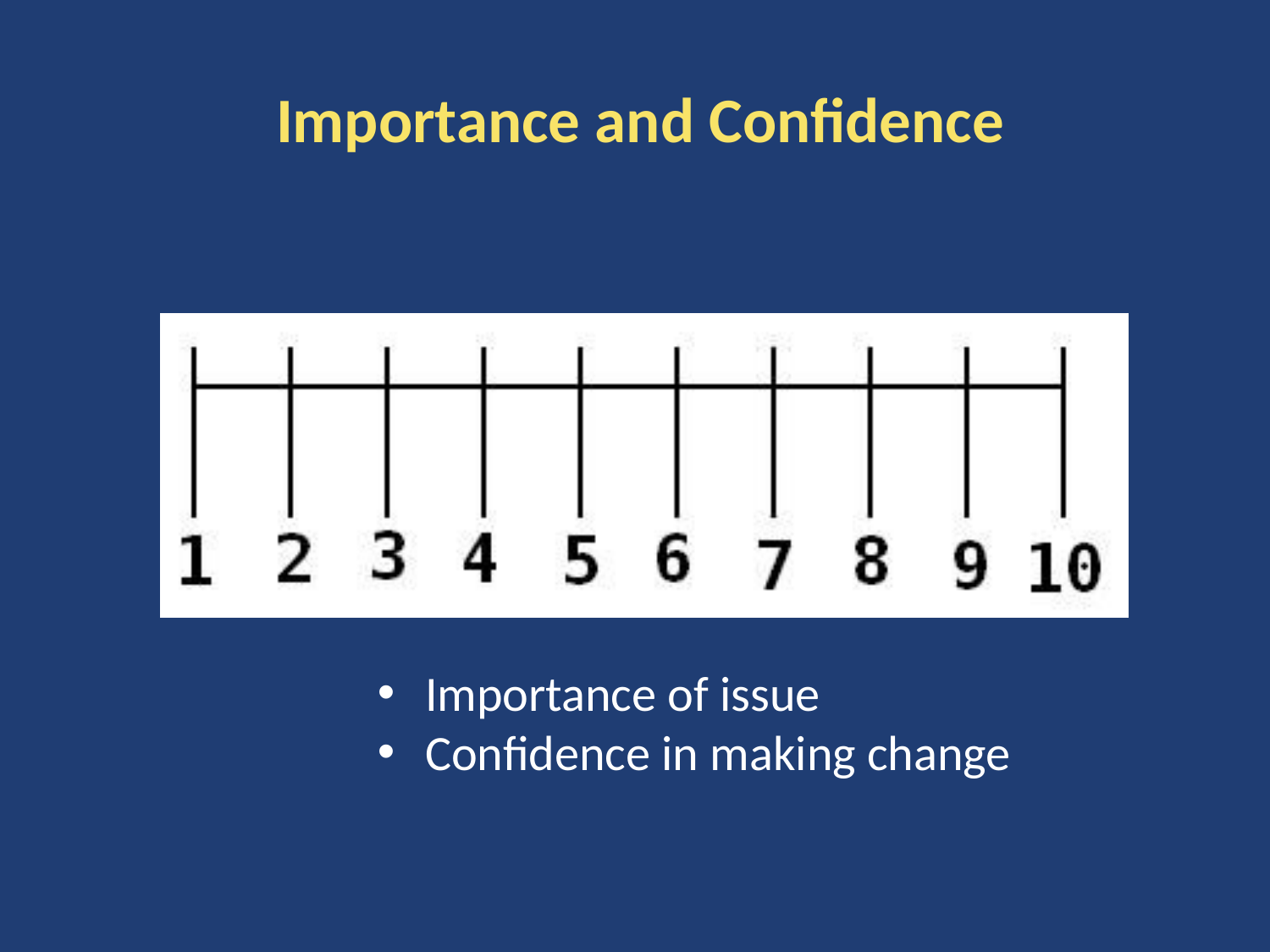

# Importance and Confidence
Importance of issue
Confidence in making change

## Slide 18
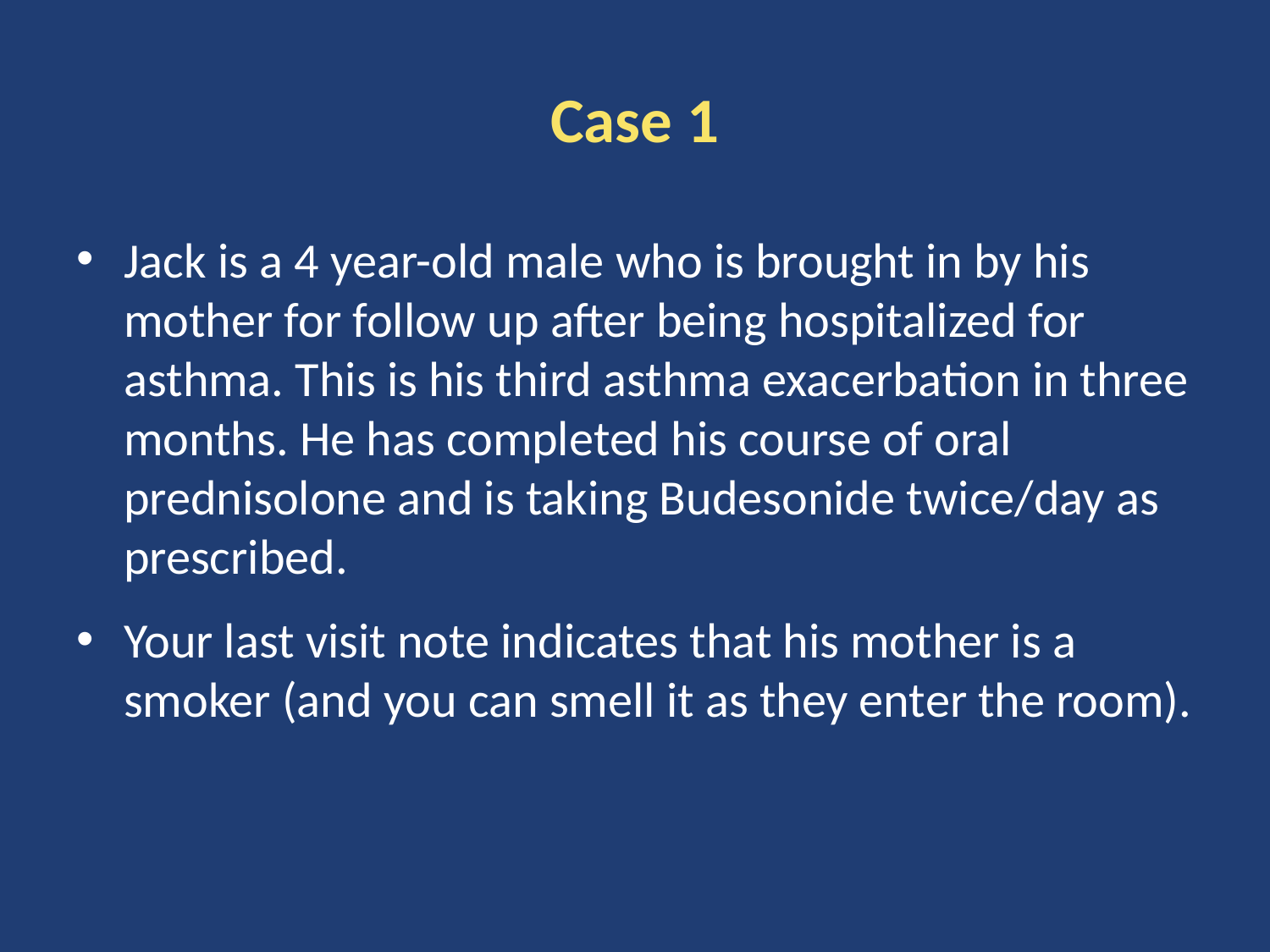

# Case 1
Jack is a 4 year-old male who is brought in by his mother for follow up after being hospitalized for asthma. This is his third asthma exacerbation in three months. He has completed his course of oral prednisolone and is taking Budesonide twice/day as prescribed.
Your last visit note indicates that his mother is a smoker (and you can smell it as they enter the room).

## Slide 19
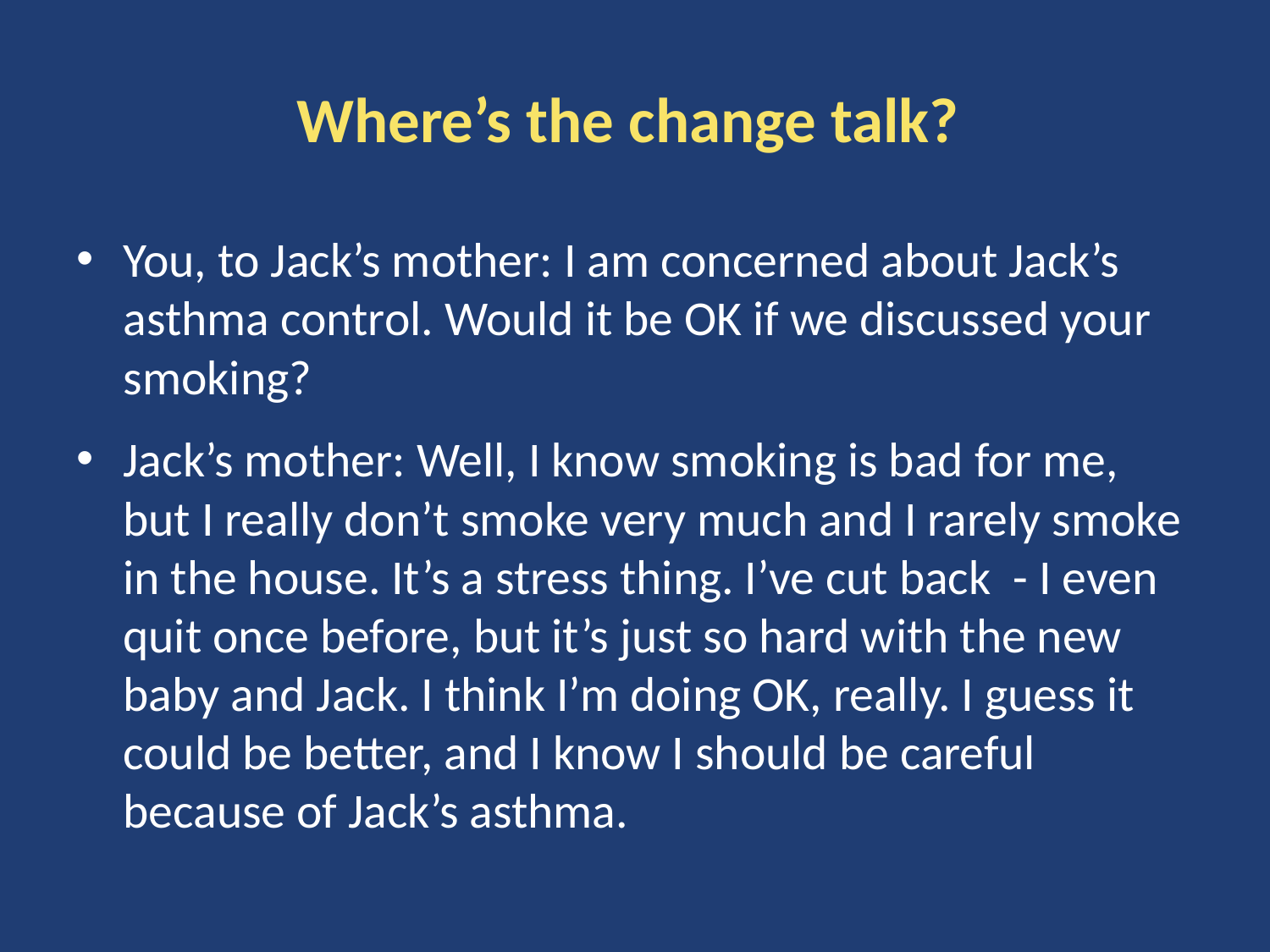

# Where’s the change talk?
You, to Jack’s mother: I am concerned about Jack’s asthma control. Would it be OK if we discussed your smoking?
Jack’s mother: Well, I know smoking is bad for me, but I really don’t smoke very much and I rarely smoke in the house. It’s a stress thing. I’ve cut back - I even quit once before, but it’s just so hard with the new baby and Jack. I think I’m doing OK, really. I guess it could be better, and I know I should be careful because of Jack’s asthma.

## Slide 20
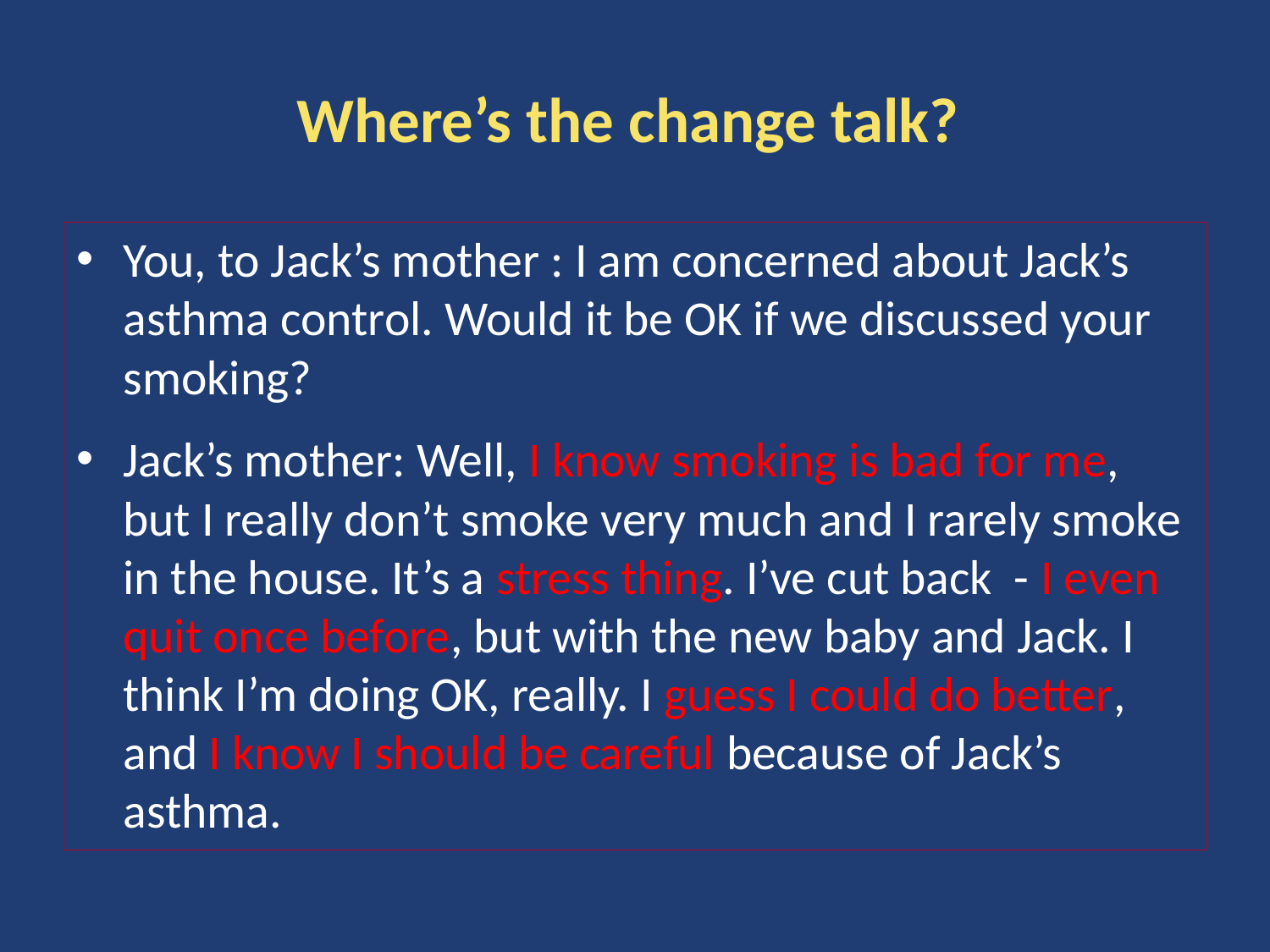

# Where’s the change talk?
You, to Jack’s mother : I am concerned about Jack’s asthma control. Would it be OK if we discussed your smoking?
Jack’s mother: Well, I know smoking is bad for me, but I really don’t smoke very much and I rarely smoke in the house. It’s a stress thing. I’ve cut back - I even quit once before, but with the new baby and Jack. I think I’m doing OK, really. I guess I could do better, and I know I should be careful because of Jack’s asthma.

## Slide 21
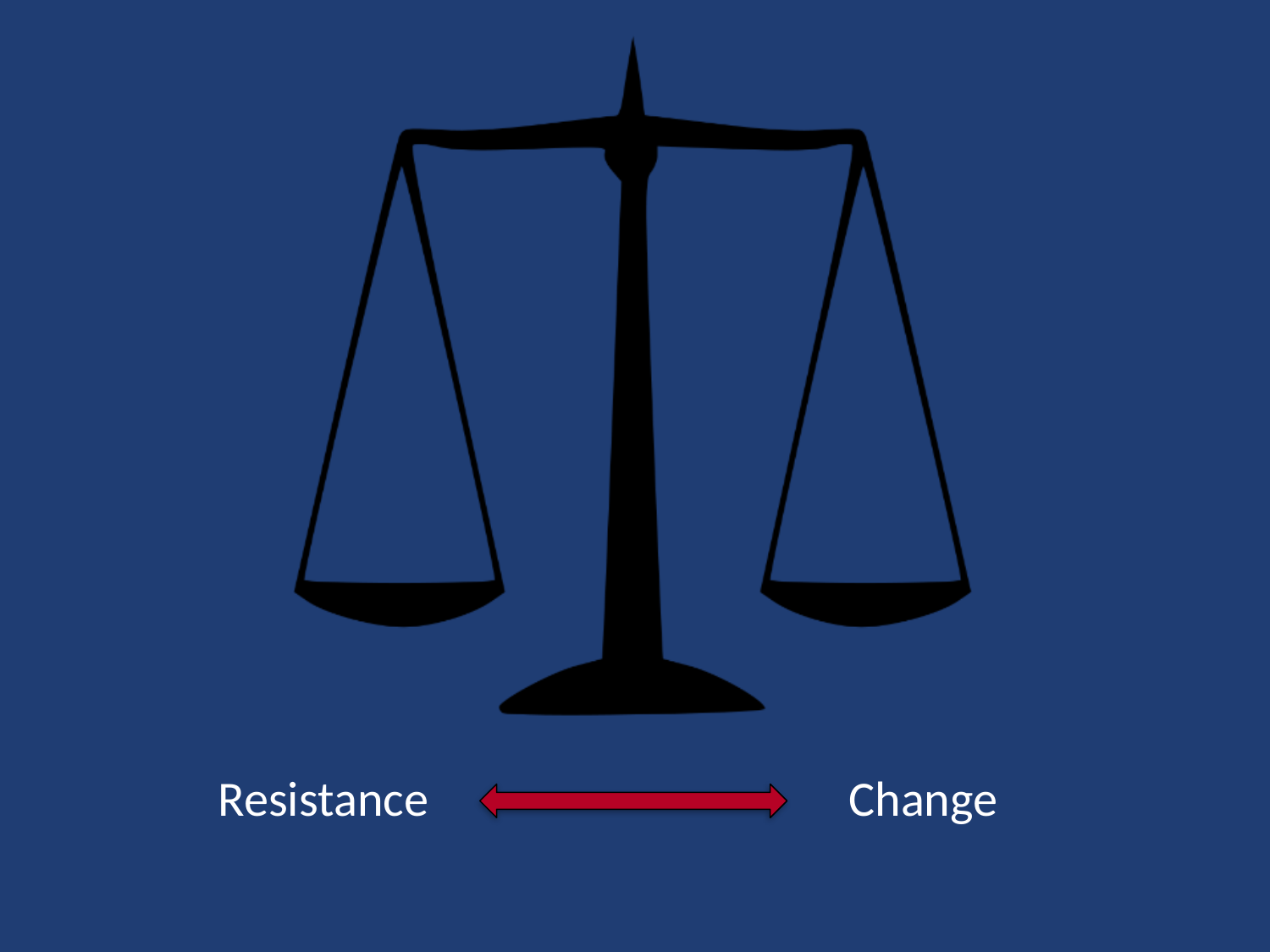

Resistance
Change

## Slide 22
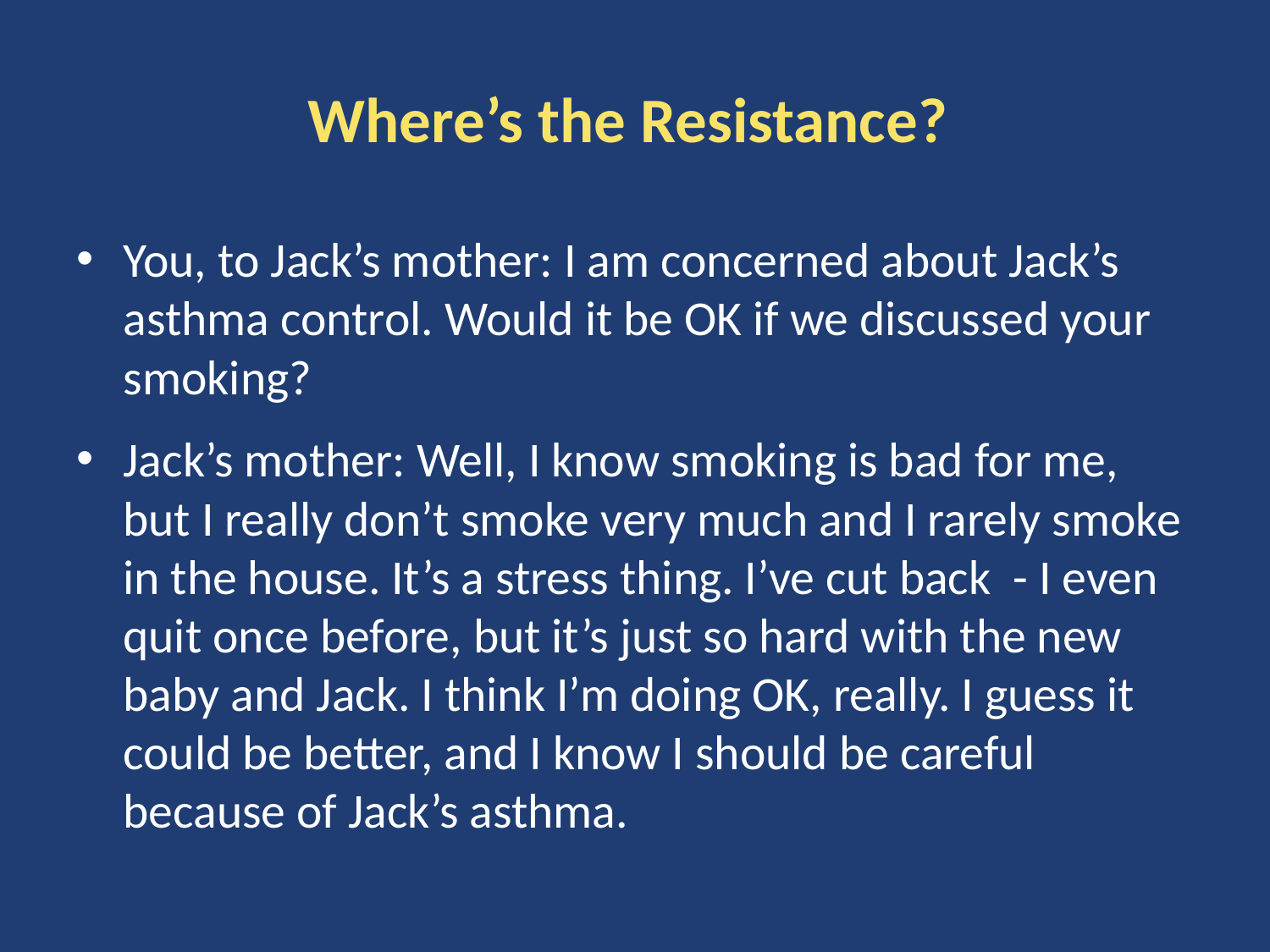

# Where’s the Resistance?
You, to Jack’s mother: I am concerned about Jack’s asthma control. Would it be OK if we discussed your smoking?
Jack’s mother: Well, I know smoking is bad for me, but I really don’t smoke very much and I rarely smoke in the house. It’s a stress thing. I’ve cut back - I even quit once before, but it’s just so hard with the new baby and Jack. I think I’m doing OK, really. I guess it could be better, and I know I should be careful because of Jack’s asthma.

## Slide 23
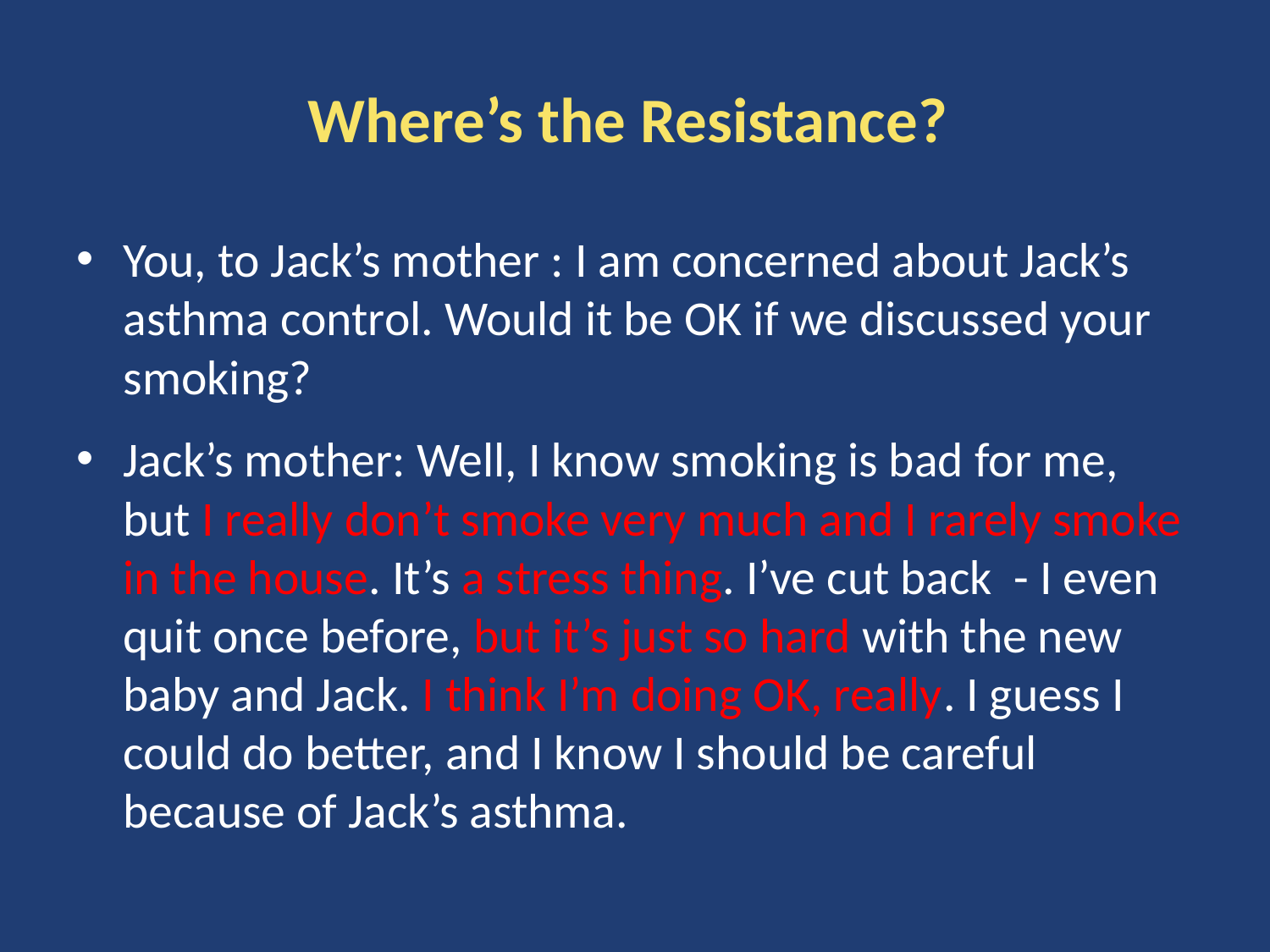

# Where’s the Resistance?
You, to Jack’s mother : I am concerned about Jack’s asthma control. Would it be OK if we discussed your smoking?
Jack’s mother: Well, I know smoking is bad for me, but I really don’t smoke very much and I rarely smoke in the house. It’s a stress thing. I’ve cut back - I even quit once before, but it’s just so hard with the new baby and Jack. I think I’m doing OK, really. I guess I could do better, and I know I should be careful because of Jack’s asthma.

## Slide 24
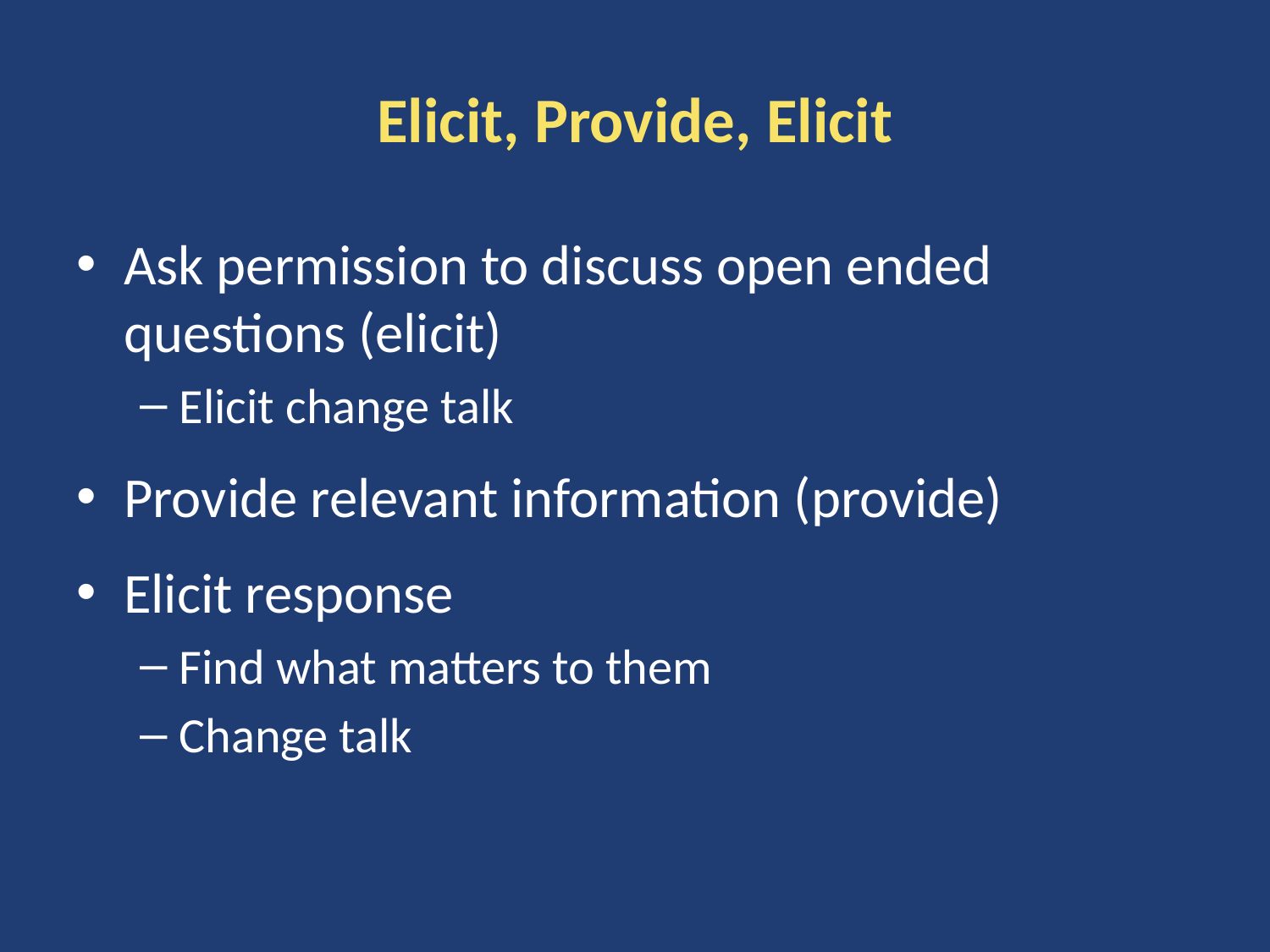

# Elicit, Provide, Elicit
Ask permission to discuss open ended questions (elicit)
Elicit change talk
Provide relevant information (provide)
Elicit response
Find what matters to them
Change talk

## Slide 25
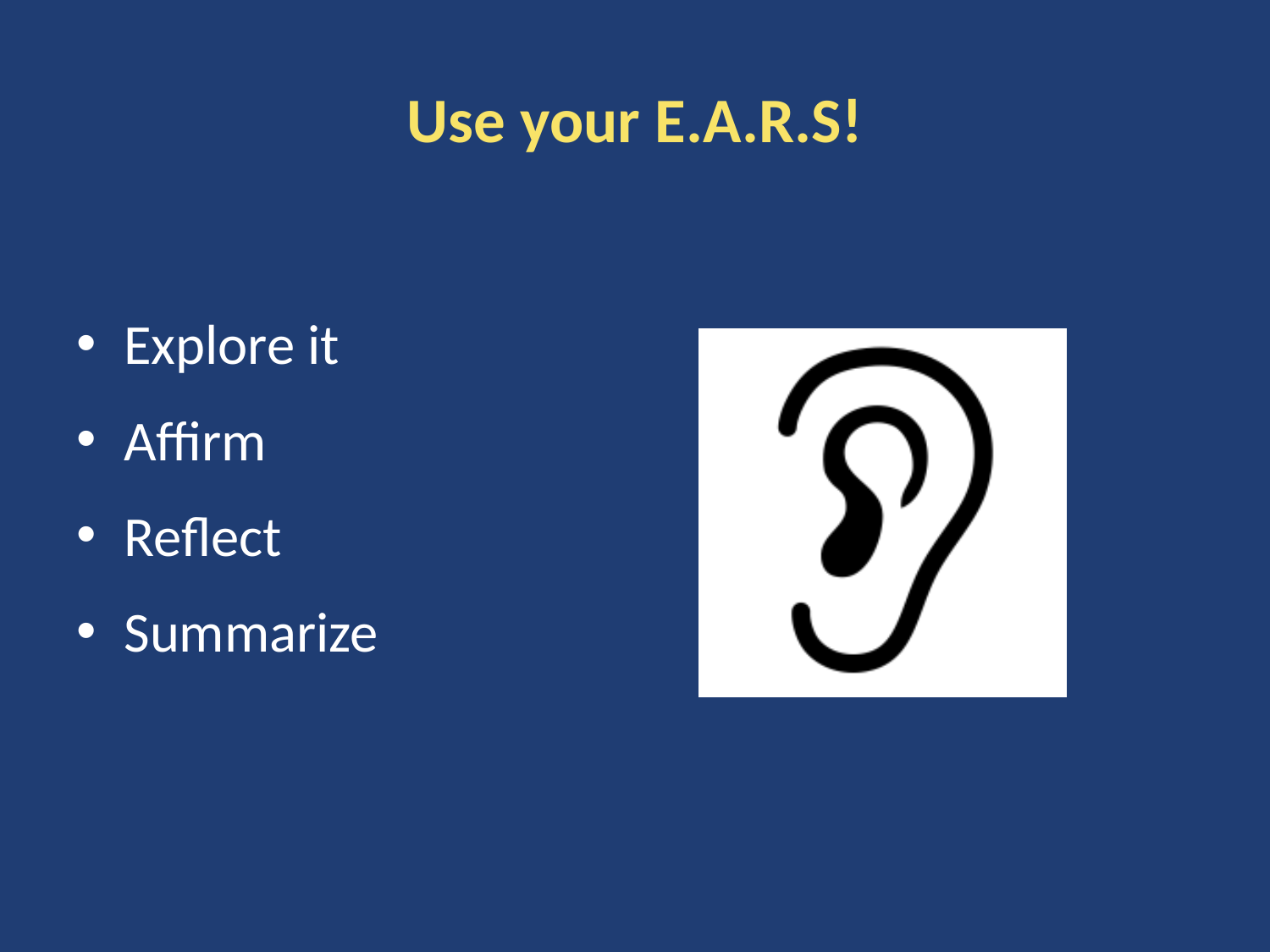

# Use your E.A.R.S!
Explore it
Affirm
Reflect
Summarize

## Slide 26
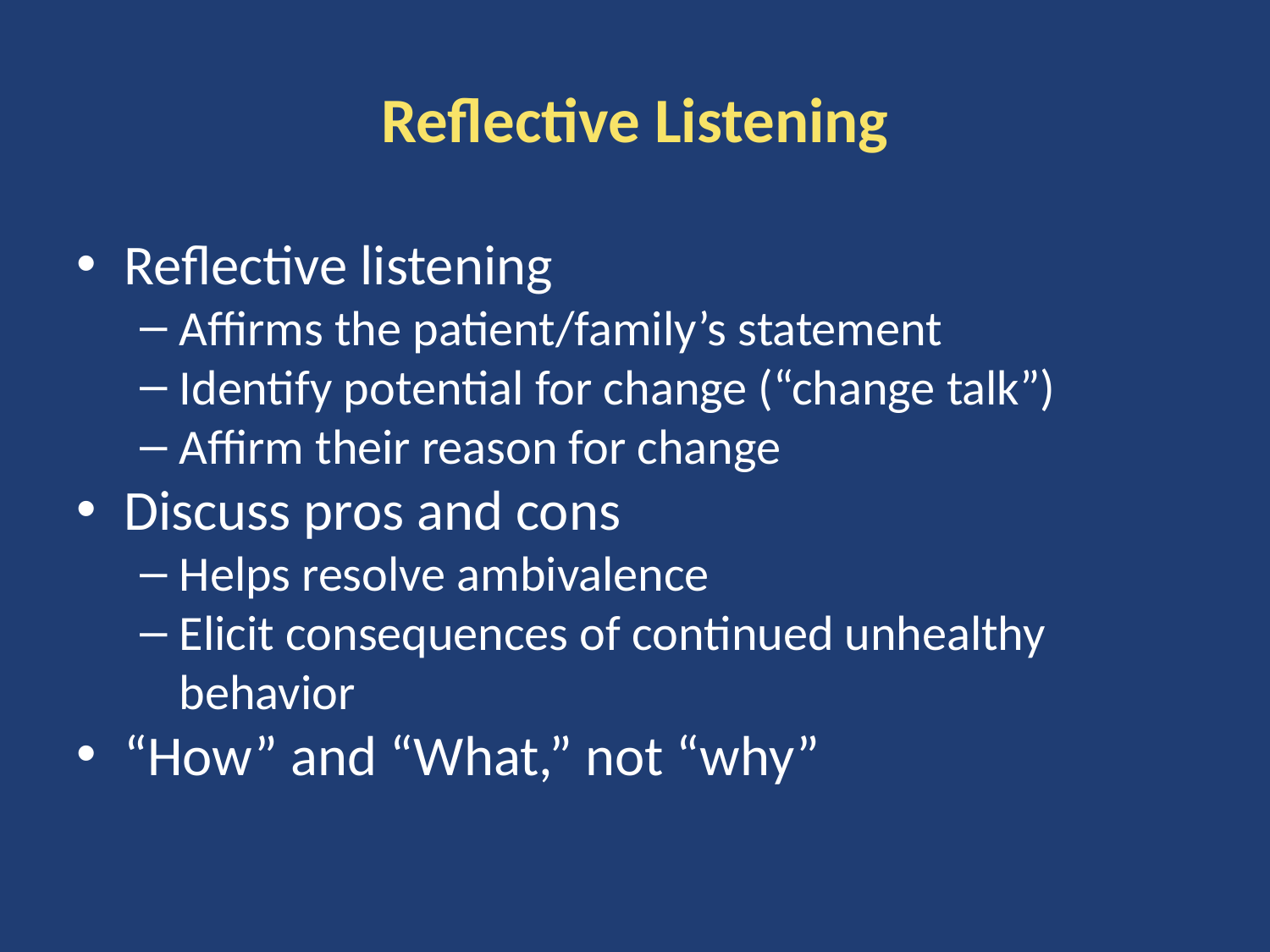

# Reflective Listening
Reflective listening
Affirms the patient/family’s statement
Identify potential for change (“change talk”)
Affirm their reason for change
Discuss pros and cons
Helps resolve ambivalence
Elicit consequences of continued unhealthy behavior
“How” and “What,” not “why”

## Slide 27
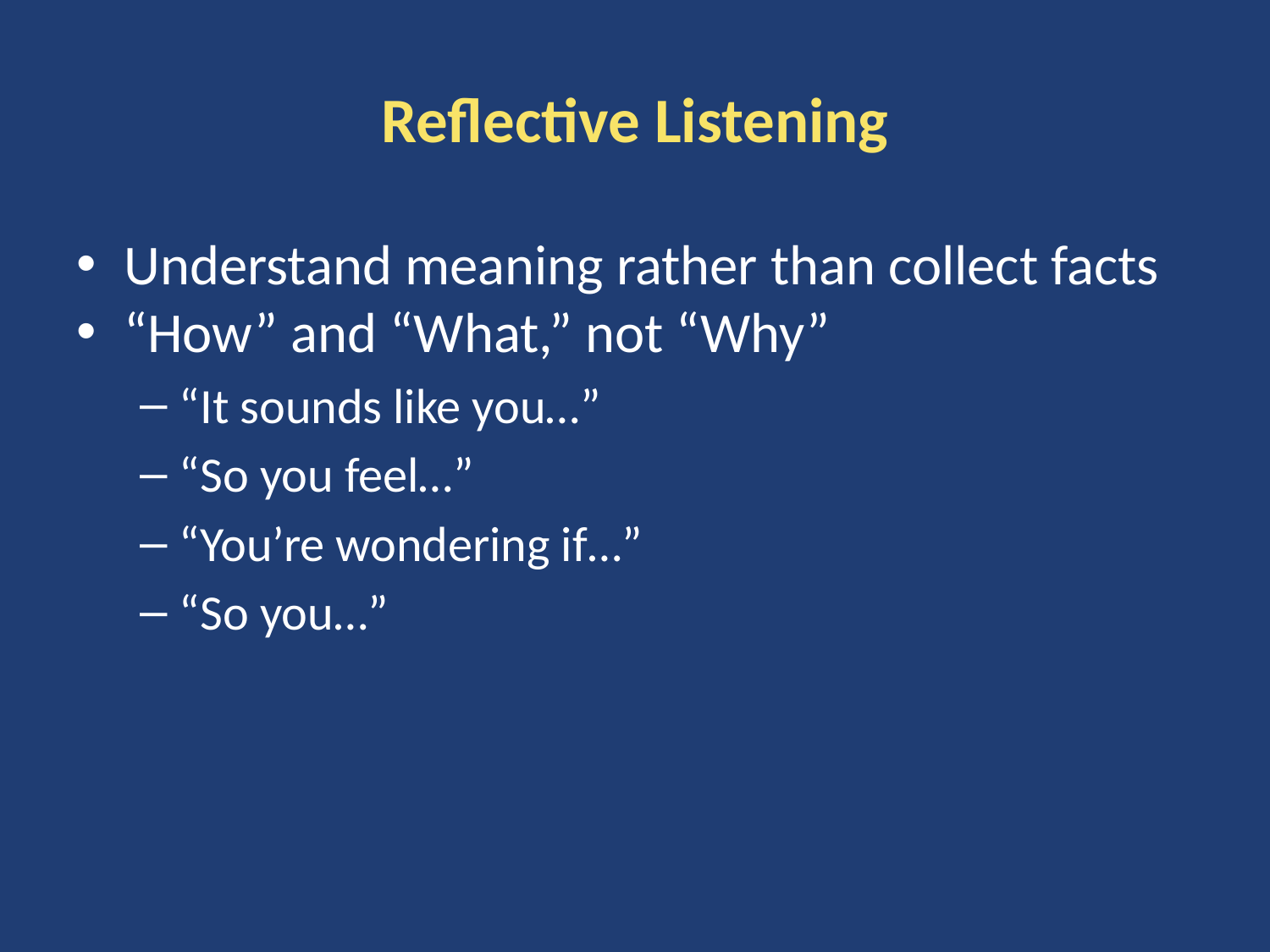

# Reflective Listening
Understand meaning rather than collect facts
“How” and “What,” not “Why”
“It sounds like you…”
“So you feel…”
“You’re wondering if…”
“So you…”

## Slide 28
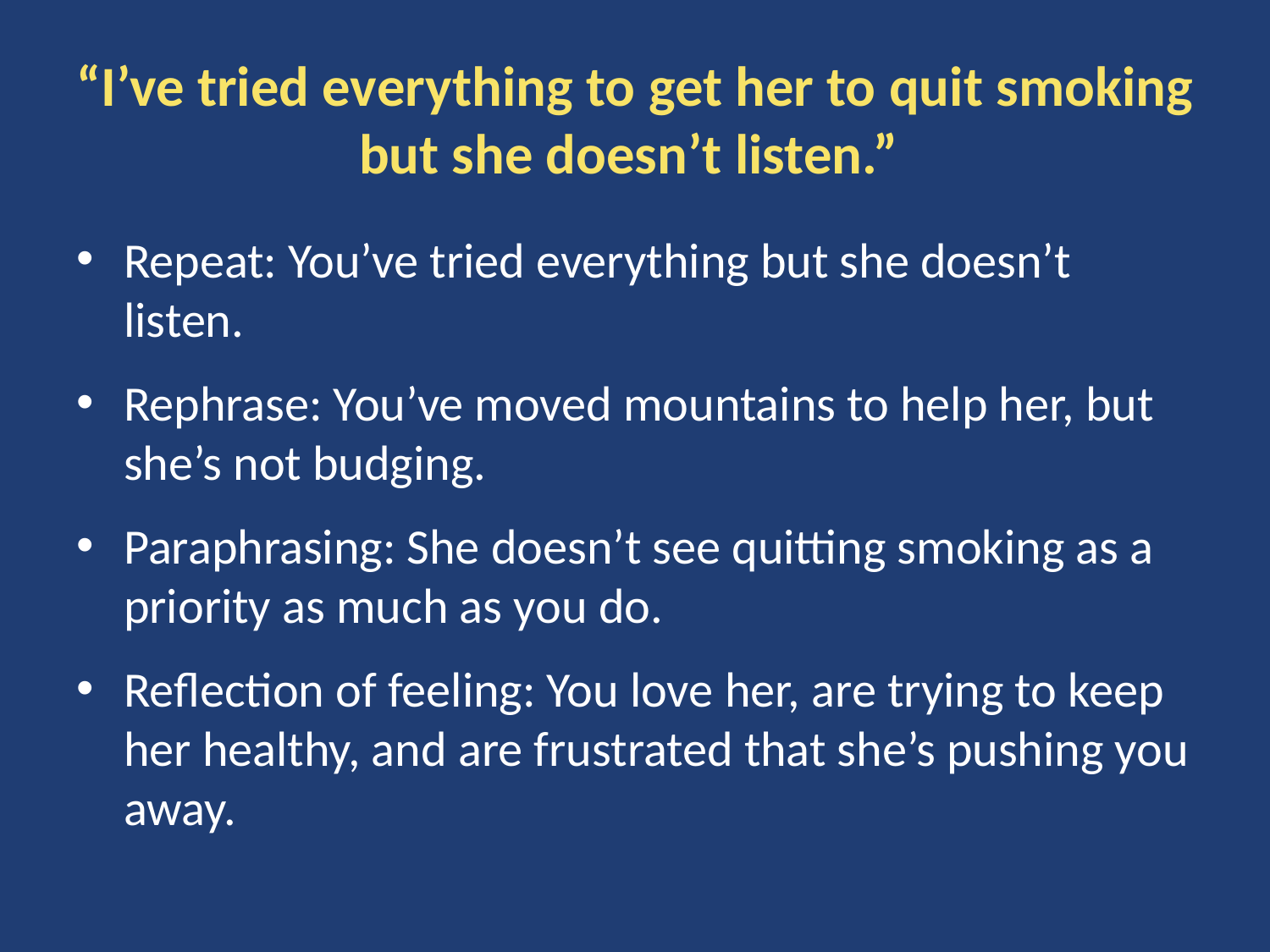

# “I’ve tried everything to get her to quit smoking but she doesn’t listen.”
Repeat: You’ve tried everything but she doesn’t listen.
Rephrase: You’ve moved mountains to help her, but she’s not budging.
Paraphrasing: She doesn’t see quitting smoking as a priority as much as you do.
Reflection of feeling: You love her, are trying to keep her healthy, and are frustrated that she’s pushing you away.

## Slide 29
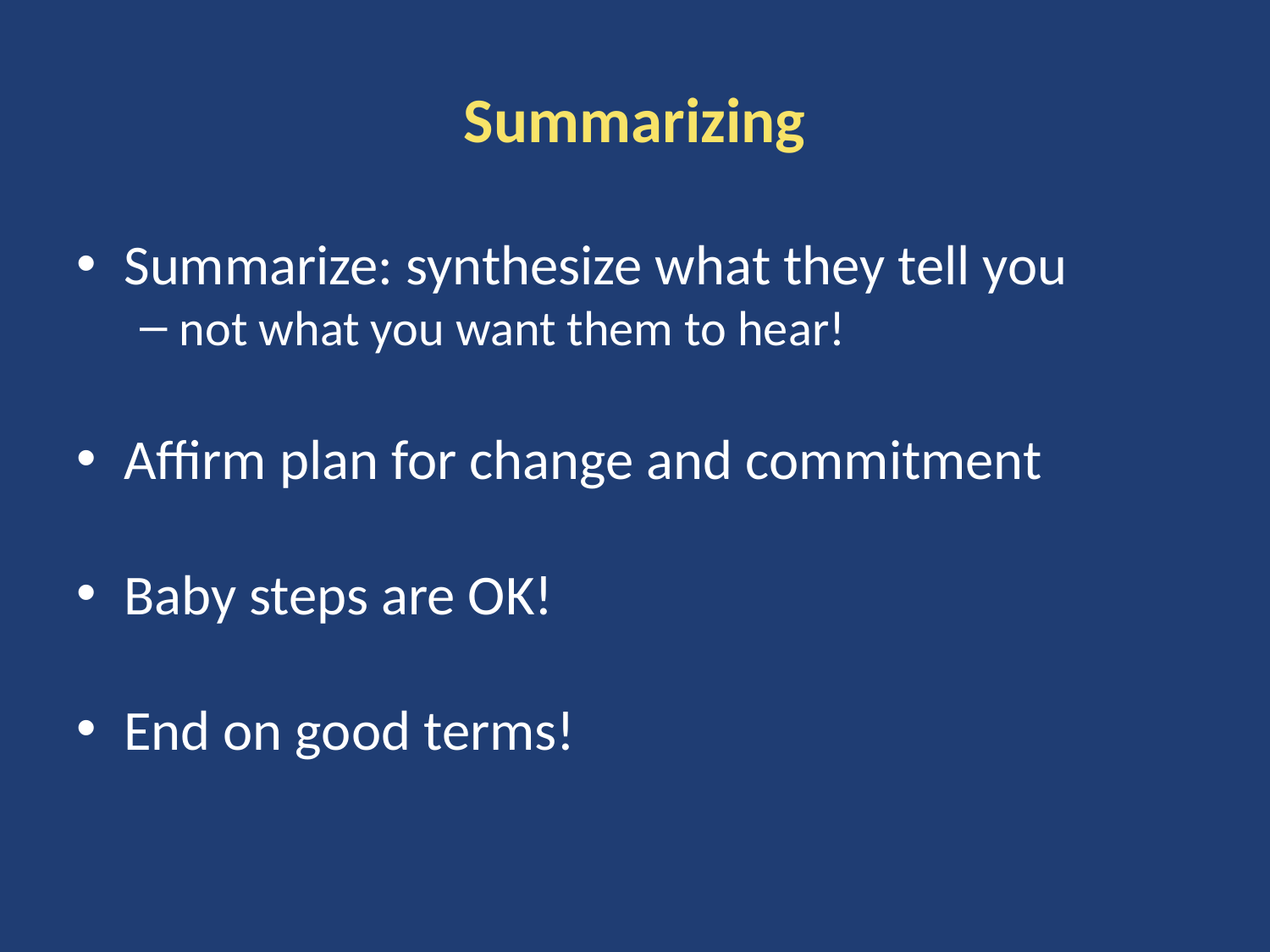

# Summarizing
Summarize: synthesize what they tell you
not what you want them to hear!
Affirm plan for change and commitment
Baby steps are OK!
End on good terms!

## Slide 30
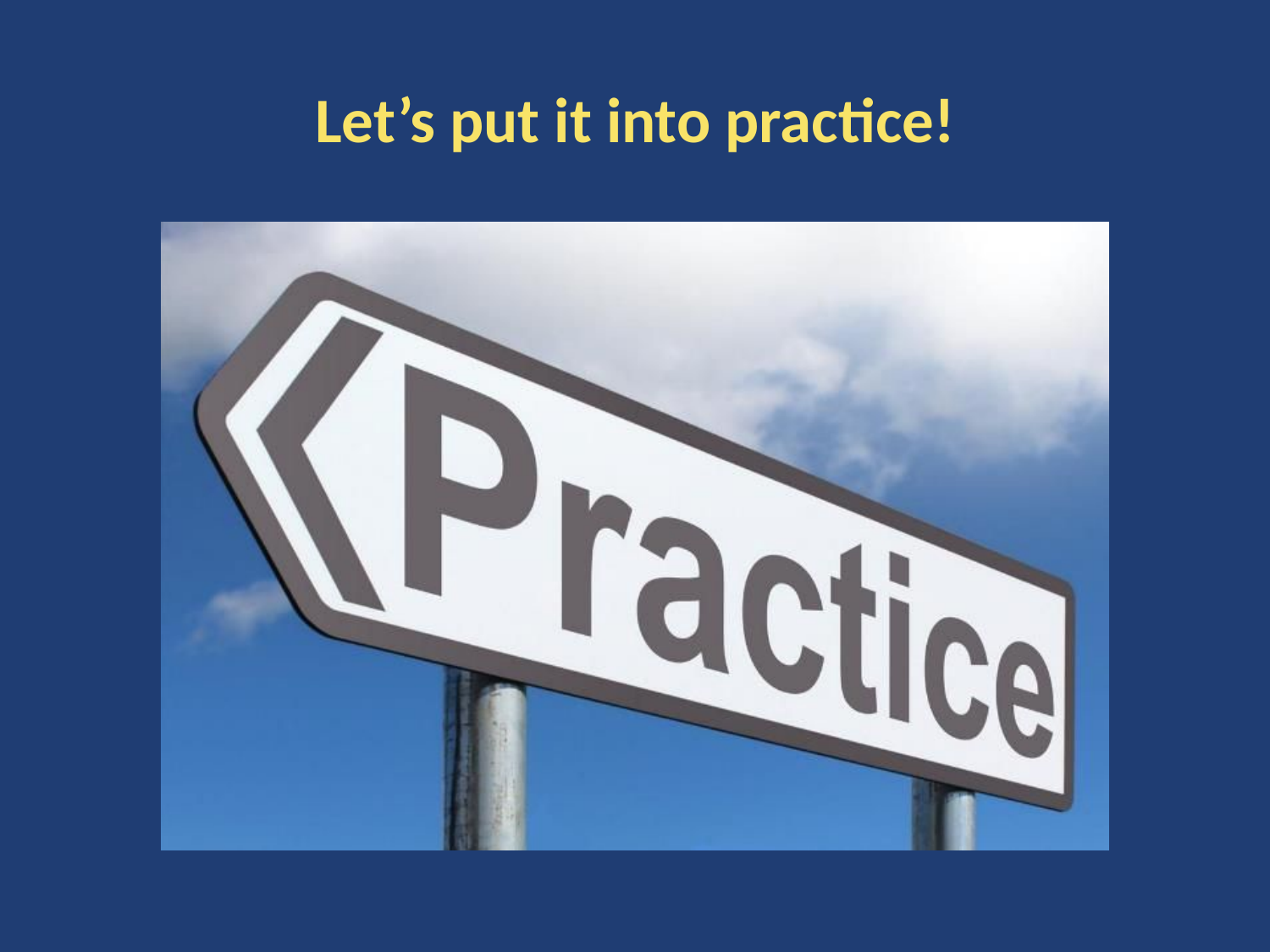

# Let’s put it into practice!
Questions?

## Slide 31
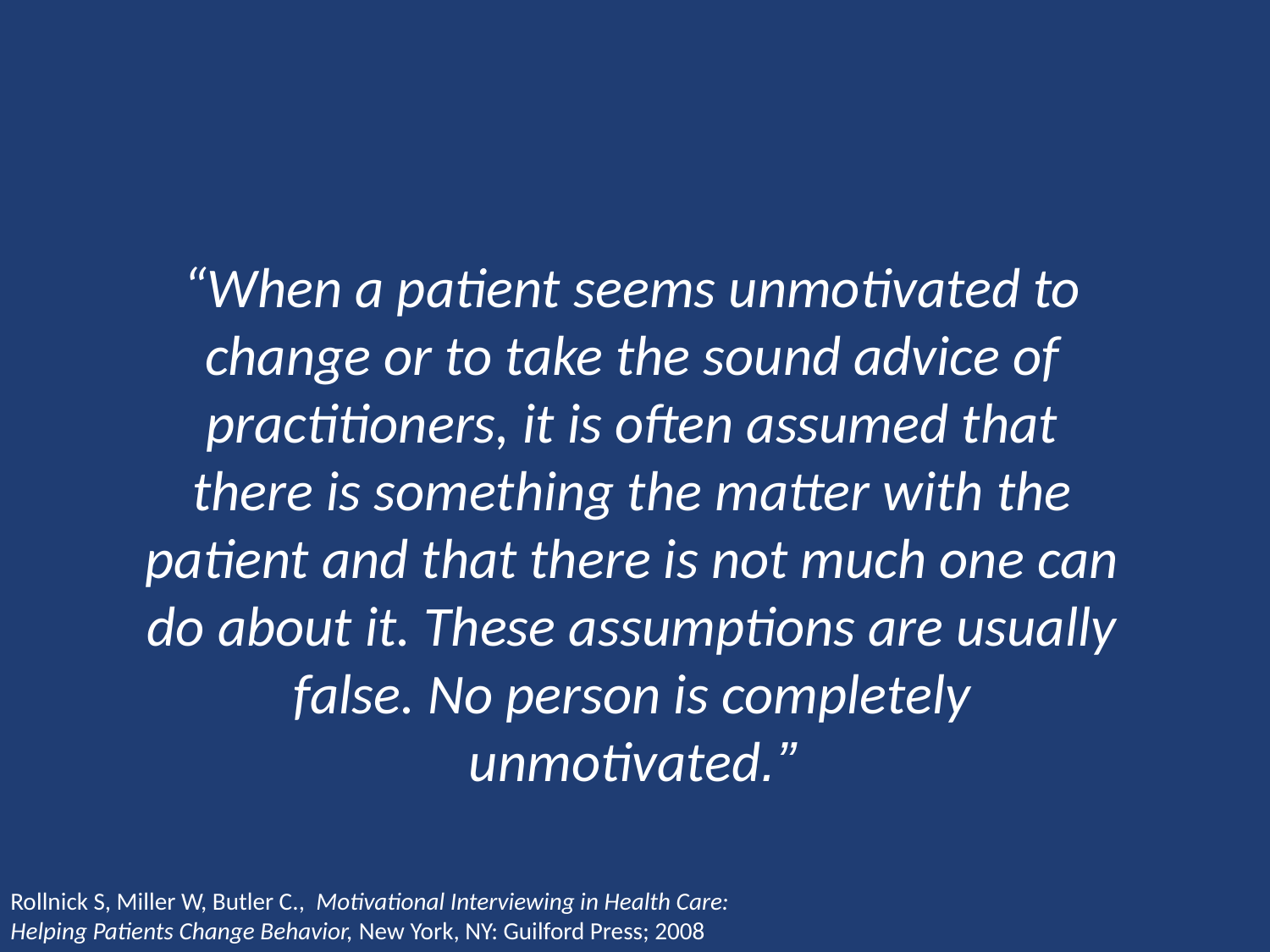

“When a patient seems unmotivated to change or to take the sound advice of practitioners, it is often assumed that there is something the matter with the patient and that there is not much one can do about it. These assumptions are usually false. No person is completely unmotivated.”
Rollnick S, Miller W, Butler C., Motivational Interviewing in Health Care:
Helping Patients Change Behavior, New York, NY: Guilford Press; 2008

## Slide 32
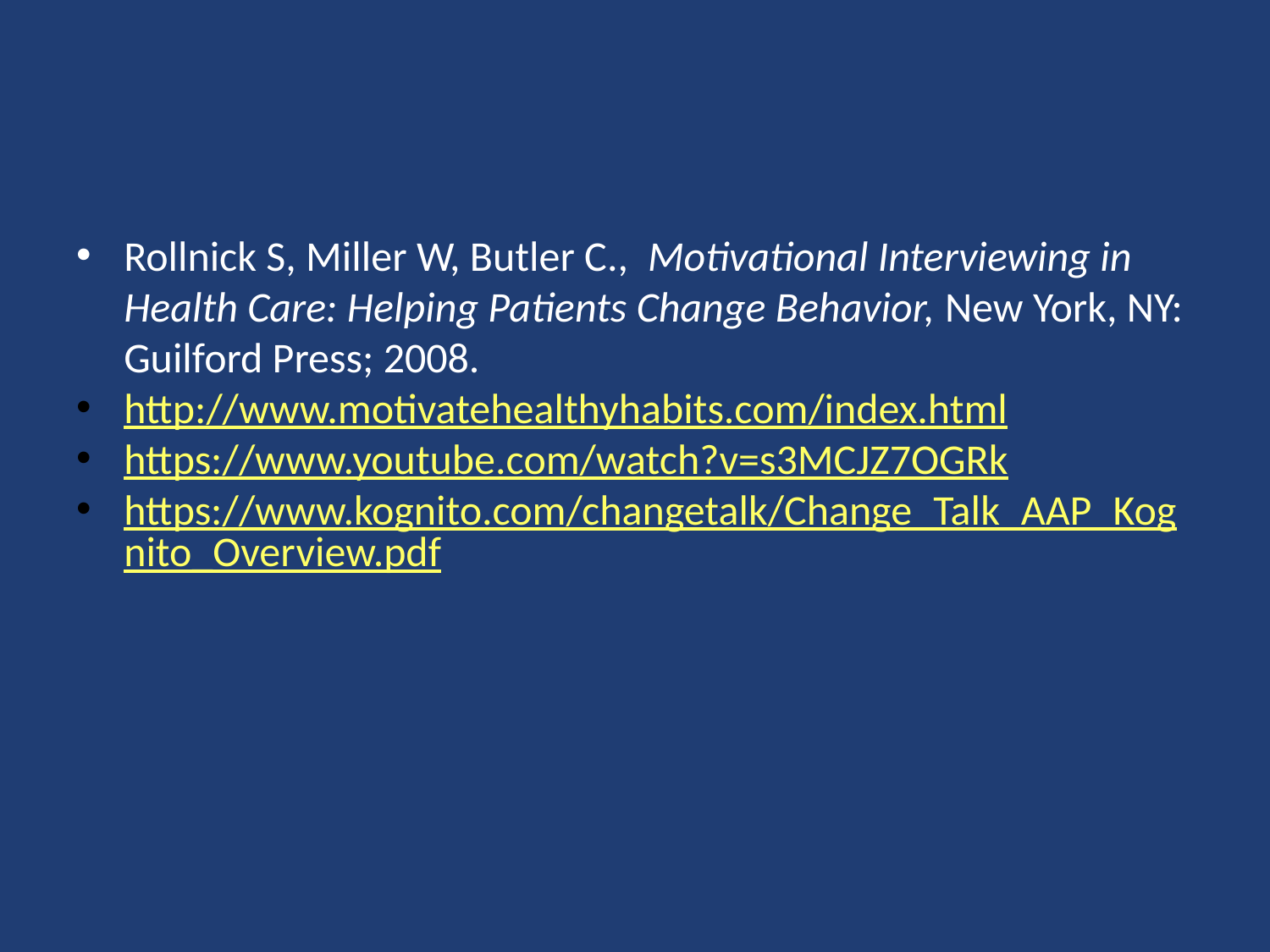

Rollnick S, Miller W, Butler C., Motivational Interviewing in Health Care: Helping Patients Change Behavior, New York, NY: Guilford Press; 2008.
http://www.motivatehealthyhabits.com/index.html
https://www.youtube.com/watch?v=s3MCJZ7OGRk
https://www.kognito.com/changetalk/Change_Talk_AAP_Kognito_Overview.pdf
