# Supplementary material for: Motivational Interviewing: A High-Yield Interactive Session for Medical Trainees and Professionals to Help Tobacco Users Quit
Source: MedEdPORTAL. 2019 Aug 23;15:10831. doi: 10.15766/mep_2374-8265.10831 (PMC6868517; doi:10.15766/mep_2374-8265.10831)
Supplement: Supplementary file 1 — A. MI Presentation.pptx B. MI Workshop Scenarios.docx C. Checklist for MI.docx D. MI Laminated Card.pptx E. Resident Survey.docx F. MI Facilitator Guide.docx [file mep-15-10831-s001.zip › B._MI_Workshop_Scenarios.docx]

**Scenario # 1**

Jack is a 4 year-old male who is brought in by his mother for follow up after being hospitalized for asthma. This is his third asthma exacerbation in three months. He has completed his course of oral prednisolone and is taking Budesonide twice/day as prescribed. Your last visit note indicates that his mother is a smoker (and you can smell it as they enter the room).

**Physician**: I am concerned about Jack’s asthma control. Would it be OK if we discussed your smoking?

**Jack’s mother**: Well, I know smoking is bad for me, but I really don’t smoke very much and I rarely smoke in the house. It’s a stress thing. I’ve cut back - I even quit once before, but it’s just so hard with the new baby and Jack. I think I’m doing OK, really. I guess it could be better, and I know I should be careful because of Jack’s asthma.

In groups of 3, continue the conversation, for 2 – 3 minutes, concentrating on some of the fundamentals of MI that we have discussed. Set the agenda in a collaborative manner, utilizing counseling skills including:

- - Reflective listening
  - Assessing readiness for change; finding change talk
  - Determine importance and confidence
  - Support development of a plan

One person will play the physician, one will play the mother, one will be observer, whose role is to identify as many of the items on the checklist as possible, and then provide feedback.

**Scenario # 2**

Emily is a 15 year-old patient you have known since she was a toddler. She is brought in today by her parents for a yearly physical. You are surprised that both parents are with her, since usually only one parent brings her. You suspect they have something they are concerned about. Indeed, Emily’s mother pulls you aside as the nurse is obtaining her vitals, saying, “Doc, we are very concerned about Emily’s behavior. We think she has been vaping and maybe also using marijuana, but we’re just not sure. We are hoping you can talk to her. Emily’s HEADDSS exam reveals that she feels safe at home, is doing well in school ninth grade and loves History, has lots of friends, but that she has started vaping a few times a week with her friends – and she has on occasion tried marijuana too.

What do you say to Emily?

In groups of 3, have a conversation with Emily, for 2 – 3 minutes, concentrating on some of the fundamentals of MI that we have discussed. Set the agenda in a collaborative manner, utilizing counseling skills including:

- - Reflective listening
  - Assessing readiness for change; finding change talk
  - Determining importance and confidence
  - Supporting development of a plan

One person will play the physician, one will play Emily, one will be observer, whose role is to identify as many of the items on the checklist as possible, and then provide feedback.

**Scenario # 3**

You are taking care of a 24 hour-old well term baby boy, Vincent, born to a 26 year-old G1P1 mother with good prenatal care and an uncomplicated pregnancy. Vincent’s mother had been a pack per day smoker when she found out she was pregnant at 10 weeks, but had cut back to 3 cigarettes/day by the end of the second trimester. Vincent’s father is also a smoker, and has also cut back.

In groups of 3, have a conversation with Vincent’s mother or father for 2 – 3 minutes, concentrating on some of the fundamentals of MI that we have discussed. Set the agenda in a collaborative manner, utilizing counseling skills including:

- - Reflective listening
  - Assessing readiness for change; finding change talk
  - Determining importance and confidence
  - Supporting development of a plan

One person will play the physician, one will play the parent, one will be observer, whose role is to identify as many of the items on the checklist as possible, and then provide feedback.

**Scenario # 4**

Andrew is an 8 year-old boy admitted to the hospital with mild intermittent asthma. He had been on a controller medication several years ago, but his pediatrician had discontinued it because his asthma was so well controlled. He has never been admitted to the hospital before. On initial exam he is mildly hypoxic (P02 91%), with RR = 36. He is able to speak in full sentences. Physical exam is significant for a generally well appearing boy with subcostal retractions, scattered wheezes and a prolonged expiratory phase. As he is taking his Albuterol, you realize you suspect that his father is a smoker, as you can smell it in the room.

In groups of 3, have a conversation with Andrew’s father, for 2 – 3 minutes, concentrating on some of the fundamentals of MI that we have discussed. Set the agenda in a collaborative manner, utilizing counseling skills including:

- - Reflective listening
  - Assessing readiness for change; finding change talk
  - Determining importance and confidence
  - Supporting development of a plan

One person will play the physician/nurse/respiratory therapist, one will play Andrew’s father, one will be observer, whose role is to identify as many of the items on the checklist as possible, and then provide feedback.

**Scenario # 5**

You are working in the Emergency Department in December. You are called to see a 14 month-old female in mild respiratory distress, brought in by her mother. PE is remarkable for a fever of 101, Pulse 130, RR 54, P02 94% on RA, BP 80/50. Lung exam is significant for subcostal retractions, fair air entry, scattered wheezes and crackles. Remainder of PE is WNL and she appears well hydrated. On reviewing the intake form you notice that she had been brought in a month ago with similar symptoms and that both parents are smokers.

In groups of 3, have a conversation with the mother, for 2 – 3 minutes, concentrating on some of the fundamentals of MI that we have discussed: Setting the agenda in a collaborative manner, utilizing counseling skills including:

- - Reflective listening
  - Assessing readiness for change; finding change talk
  - Determining importance and confidence
  - Supporting development of a plan

One person will play the physician/nurse/respiratory therapist, one will play the mother, one will be observer, whose role is to identify as many of the items on the checklist as possible, and then provide feedback.

**Scenario # 6**

You are working on a busy inpatient service, caring for a 10 year-old girl, Anna, with newly diagnosed Crohn’s disease. Her parents seem very stressed, though Anna herself seems to be handling this new diagnosis well. Anna’s parents leave the hospital frequently to go smoke, which upsets Anna. You want to talk to her parents about smoking, but aren’t quite sure how to start the conversation.

In groups of 3, have a conversation with Anna’s mother or father, for 2 – 3 minutes, concentrating on some of the fundamentals of MI that we have discussed: Setting the agenda in a collaborative manner, utilizing counseling skills including:

- - Reflective listening
  - Assessing readiness for change; finding change talk
  - Determining importance and confidence
  - Supporting development of a plan

One person will play the physician/nurse/respiratory therapist, one will play the mother, one will be observer, whose role is to identify as many of the items on the checklist as possible, and then provide feedback.

**Scenario # 7**

You are working in the NICU caring for several critical babies on ventilators. Over the course of your busy week you realize that the mother of one of the babies has been leaving to smoke every afternoon (despite the hospital’s no smoking policy). You ask a co-worker if anyone has spoken with the mother about her smoking, but your colleague just sighs and says, “I don’t know. She shouldn’t be smoking but I’m not going to confront her on that.” What do you say to your co-worker? What do you say to the mother?

In groups of 3, have a conversation with the co-worker or with the mother. In your conversation with the mother, take 2 – 3 minutes, concentrating on some of the fundamentals of MI that we have discussed: Setting the agenda in a collaborative manner, utilizing counseling skills including:

- - Reflective listening
  - Assessing readiness for change; finding change talk
  - Determining importance and confidence
  - Supporting development of a plan

One person will play the physician/nurse/respiratory therapist, one will play the mother, one will be observer, whose role is to identify as many of the items on the checklist as possible, and then provide feedback.
