# Supplementary material for: Motivational Interviewing: A High-Yield Interactive Session for Medical Trainees and Professionals to Help Tobacco Users Quit
Source: MedEdPORTAL. 2019 Aug 23;15:10831. doi: 10.15766/mep_2374-8265.10831 (PMC6868517; doi:10.15766/mep_2374-8265.10831)
Supplement: Supplementary file 1 — A. MI Presentation.pptx B. MI Workshop Scenarios.docx C. Checklist for MI.docx D. MI Laminated Card.pptx E. Resident Survey.docx F. MI Facilitator Guide.docx [file mep-15-10831-s001.zip › C._Checklist_for_MI.docx]

**Checklist for MI**

*This checklist is to be used by the observer in each group, and should serve as a starting point for discussion after each scenario. As you watch the scenario, pay attention to whether your colleague playing the counselor utilizes any of the following MI-specific skills. You may circle the items, or notate beside them. There is no official scoring for this checklist. Were there MI techniques noted below that your colleague did not implement? If so, could you provide some feedback as to how she might have done so?*

| MI Principle | Used? (Y/N) | Comments |
| --- | --- | --- |
| Open-ended question |  |  |
| Agenda setting |  |  |
| Explore ambivalence; assess readiness for change |  |  |
| Resist the righting reflex |  |  |
| Understand smoker’s motivation |  |  |
| Listen reflectively |  |  |
| Express empathy |  |  |
| Informing/providing information |  |  |
| Directing style |  |  |
| Guiding style |  |  |
| Following style |  |  |
