# Supplementary material for: Motivational Interviewing: A High-Yield Interactive Session for Medical Trainees and Professionals to Help Tobacco Users Quit
Source: MedEdPORTAL. 2019 Aug 23;15:10831. doi: 10.15766/mep_2374-8265.10831 (PMC6868517; doi:10.15766/mep_2374-8265.10831)
Supplement: Supplementary file 1 — A. MI Presentation.pptx B. MI Workshop Scenarios.docx C. Checklist for MI.docx D. MI Laminated Card.pptx E. Resident Survey.docx F. MI Facilitator Guide.docx [file mep-15-10831-s001.zip › D._MI_Laminated_Card.pptx]

## Slide 1
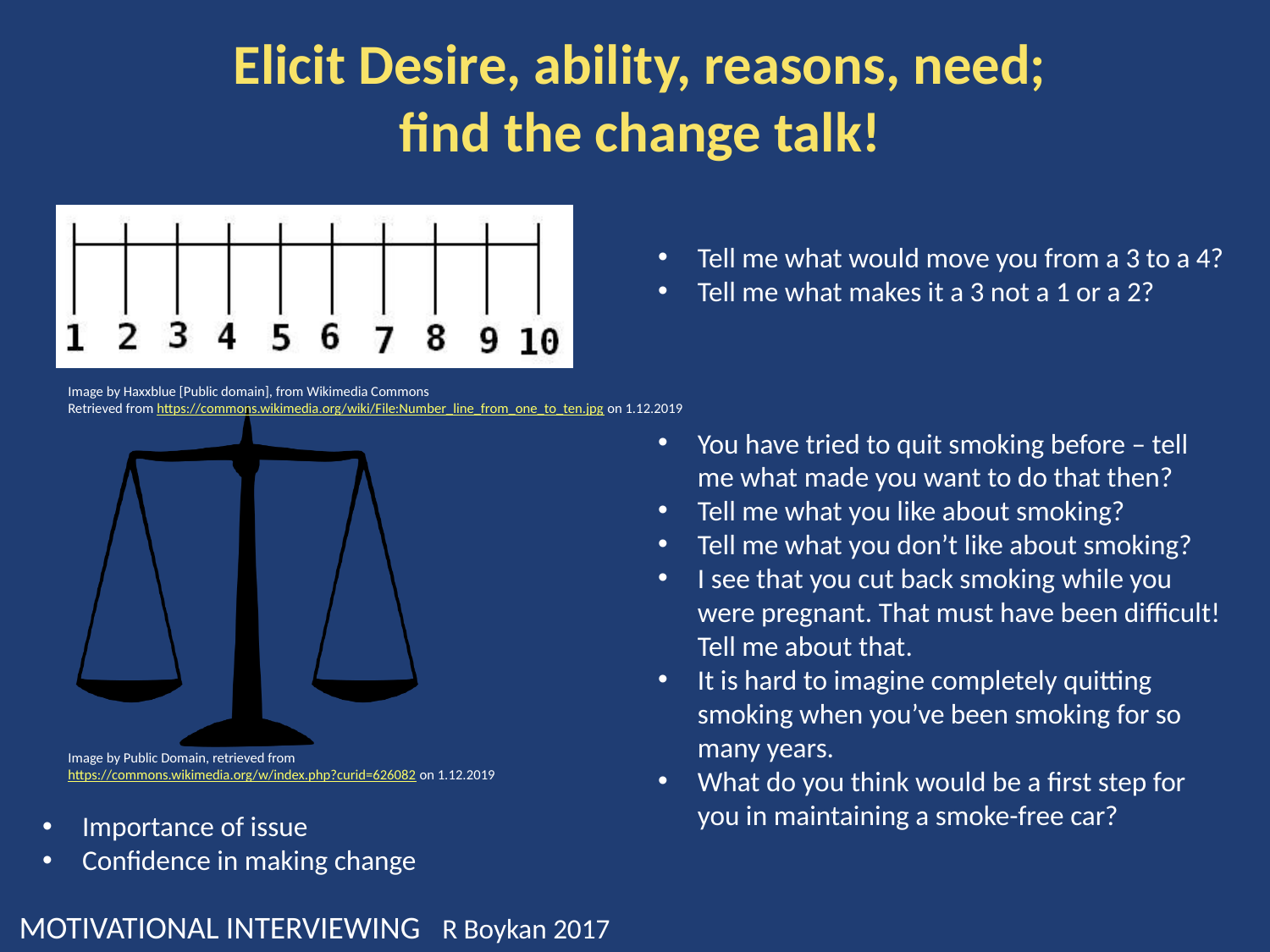

# Elicit Desire, ability, reasons, need; find the change talk!
Tell me what would move you from a 3 to a 4?
Tell me what makes it a 3 not a 1 or a 2?
Image by Haxxblue [Public domain], from Wikimedia Commons
Retrieved from https://commons.wikimedia.org/wiki/File:Number_line_from_one_to_ten.jpg on 1.12.2019
You have tried to quit smoking before – tell me what made you want to do that then?
Tell me what you like about smoking?
Tell me what you don’t like about smoking?
I see that you cut back smoking while you were pregnant. That must have been difficult! Tell me about that.
It is hard to imagine completely quitting smoking when you’ve been smoking for so many years.
What do you think would be a first step for you in maintaining a smoke-free car?
Image by Public Domain, retrieved from https://commons.wikimedia.org/w/index.php?curid=626082 on 1.12.2019
Importance of issue
Confidence in making change
MOTIVATIONAL INTERVIEWING R Boykan 2017

## Slide 2
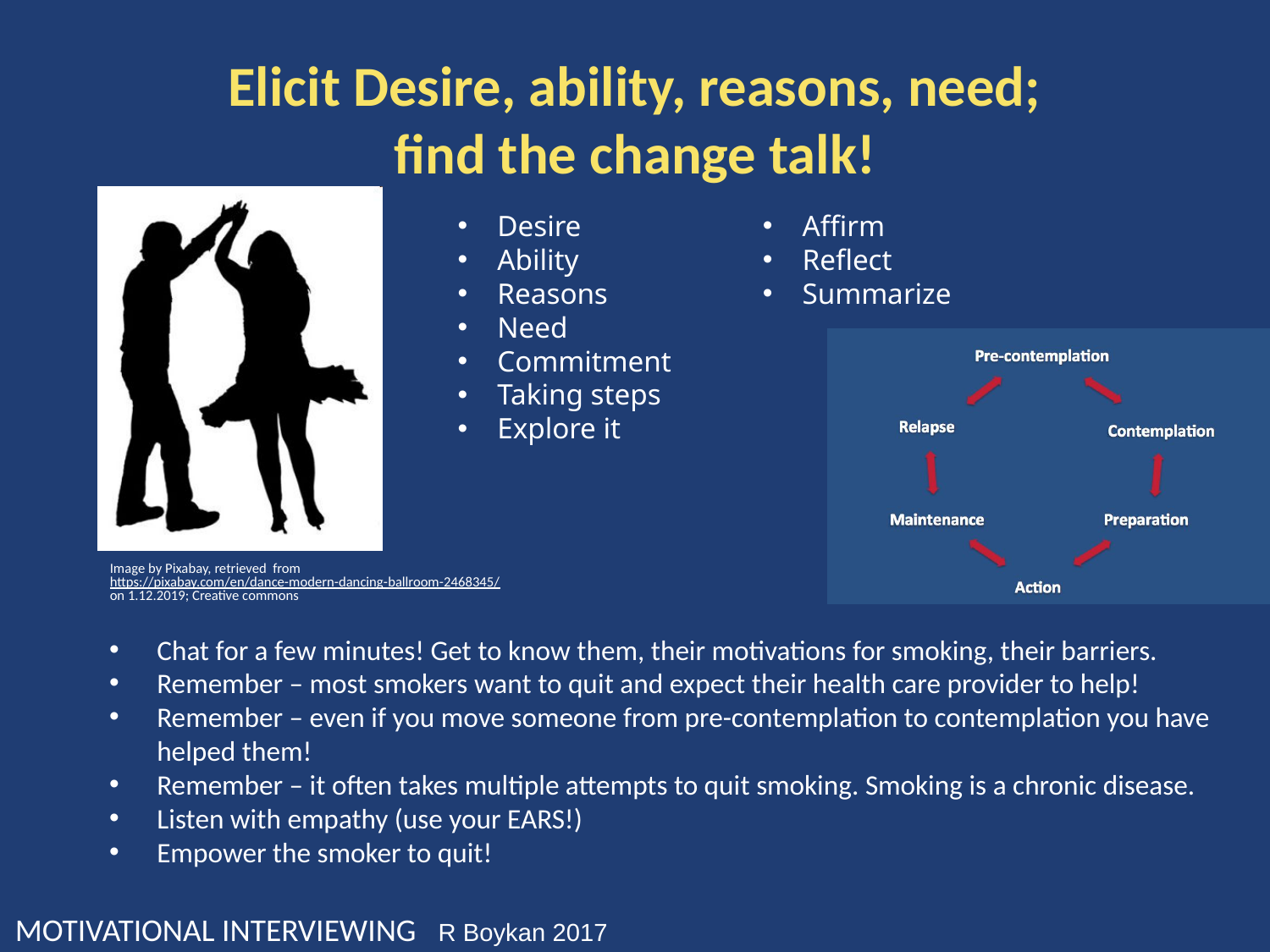

# Elicit Desire, ability, reasons, need; find the change talk!
Desire
Ability
Reasons
Need
Commitment
Taking steps
Explore it
Affirm
Reflect
Summarize
Image by Pixabay, retrieved from
https://pixabay.com/en/dance-modern-dancing-ballroom-2468345/
on 1.12.2019; Creative commons
Chat for a few minutes! Get to know them, their motivations for smoking, their barriers.
Remember – most smokers want to quit and expect their health care provider to help!
Remember – even if you move someone from pre-contemplation to contemplation you have helped them!
Remember – it often takes multiple attempts to quit smoking. Smoking is a chronic disease.
Listen with empathy (use your EARS!)
Empower the smoker to quit!
MOTIVATIONAL INTERVIEWING R Boykan 2017
