# Supplementary material for: Motivational Interviewing: A High-Yield Interactive Session for Medical Trainees and Professionals to Help Tobacco Users Quit
Source: MedEdPORTAL. 2019 Aug 23;15:10831. doi: 10.15766/mep_2374-8265.10831 (PMC6868517; doi:10.15766/mep_2374-8265.10831)
Supplement: Supplementary file 1 — A. MI Presentation.pptx B. MI Workshop Scenarios.docx C. Checklist for MI.docx D. MI Laminated Card.pptx E. Resident Survey.docx F. MI Facilitator Guide.docx [file mep-15-10831-s001.zip › E._Resident_Survey.docx]

1. Name or ID: _______________
2. What is your current level of training in residency?
   1. PGY-1
   2. PGY-2
   3. PGY-3
   4. PGY-4
3. How do you identify yourself?
   1. Male
   2. Female
   3. Transgender
   4. Neither male/female/transgender
4. Have you ever smoked cigarettes, or other tobacco products (cigars, cigareillos, pipes, etc)?
   1. Yes, I currently smoke
   2. I do not smoke now but I have smoked in the past
   3. No, I have never smoked
5. As a health professional, I feel it is my responsibility to address my patients' smoking.
   1. Agree
   2. Disagree
   3. Not sure
6. As a health professional, I feel it is my responsibility to address my patients' family members' smoking.
   1. Agree
   2. Disagree
   3. Not sure
7. I assist my patients/patients' families in their efforts to stop using tobacco products:
   1. All of the time
   2. Some of the time
   3. Never
8. If some of the time or never, why? Choose all that apply
   1. Lack of time
   2. Lack of resources
   3. Lack of reimbursement
   4. Uncomfortable discussion
   5. N/A
   6. Other (please specify) ________________
9. If a patient/family member is a smoker I refer him/her to the State Smokers' Quitline
   1. All of the time
   2. Some of the time
   3. Never
10. If some of the time or never, why? Choose all that apply.
    1. Lack of time
    2. I forget to do it
    3. Don't know how to access the state Quitline
    4. Uncomfortable discussion
    5. N/A
    6. Other (please specify)
11. The majority of smokers want to quit
    1. Agree
    2. Disagree
    3. Don’t know
12. Smokers expect health care workers to address their smoking
    1. Agree
    2. Disagree
    3. Don't know
13. Counseling patients in the office will improve a smoker’s quit attempts
    1. Agree
    2. Disagree
    3. Don't know
14. Asking about smoking in the office improves a smoker’s quit attempts
    1. Agree
    2. Disagree
    3. Don't know
15. In addressing tobacco smoking, AAR stands for:
    1. A:
    2. A:
    3. R:
16. Please give your best definition of Motivational Interviewing ____________
17. In Motivational Interviewing DARN stands for:
    1. D:
    2. A:
    3. R:
    4. N:
18. 18. In Motivational Interviewing RULE stands for:
    1. R:
    2. U:
    3. L:
    4. E:

*Thank you for your participation!*
