# Supplementary material for: Motivational Interviewing: A High-Yield Interactive Session for Medical Trainees and Professionals to Help Tobacco Users Quit
Source: MedEdPORTAL. 2019 Aug 23;15:10831. doi: 10.15766/mep_2374-8265.10831 (PMC6868517; doi:10.15766/mep_2374-8265.10831)
Supplement: Supplementary file 1 — A. MI Presentation.pptx B. MI Workshop Scenarios.docx C. Checklist for MI.docx D. MI Laminated Card.pptx E. Resident Survey.docx F. MI Facilitator Guide.docx [file mep-15-10831-s001.zip › F._MI_Facilitator_Guide.docx]

**Motivational Interviewing: A High-Yield Interactive Session for Medical Trainees and Professionals to Help Tobacco Users Quit**

**Instructor Guide**

**Session Time**

The session should take one hour for the initial didactic introduction (Power point) and small group practice. Recommended timeline is as follows:

Table: Recommended session timeline

| 15 minutes | Power point presentation (Appendix A) |
| --- | --- |
| 35 minutes | Facilitated role-play in groups of 3-4. Choose from 7 cases (Each person should play the counselor once). Role-play should take 2 -3 minutes with 3-6 minutes for debriefing and facilitated peer feedback. (Appendices B, C, D) |
| 10 minutes | Group Discussion: Discuss difficult encounters, solutions, lessons learned |

**Detailed Plan:**

Set up: The session should take place in a comfortable room with access to a computer and instructional projector. This session can be done with a variable number of participants, in groups of 3 (or 4 if there is an extra person).

Handouts: Each participant should be given Appendix B (scenarios), Appendix C (Checklist) and Appendix D (Laminated card). If desired, the survey (Appendix E) can be given at any time prior to and following the session.

**Facilitator Qualifications and Responsibilities**

- The facilitator should have knowledge of MI principles and experience utilizing MI within the clinical context of patient interactions. S/he should feel comfortable providing guidance and feedback to participants during the small-group role-play scenarios. Individuals need not have advanced qualifications in MI, however. Review of Rollnick and Miller’s book and other resources listed at the end of this document are useful prior to leading the session.
- The facilitator should have reviewed all the materials, including familiarizing himself with all the scenarios.
- The facilitator should anticipate and be comfortable answering questions that might arise during the session, and should be able to recognize and model MI principles in dialogue. For example, she should be able to provide an example for reflective listening or recognize the righting reflex (and provide an alternative way of interacting).
- The instructor should be comfortable facilitating a small group of students in self-directed learning.

**Facilities**

- Room of adequate size for group with enough space and chairs for small groups of 3 to work independently.
- Computer with instructor projector

**Tips for Success with this Curricular Module**

- The Power point should take 15 minutes. The facilitator should be aware that if the group has a significant number of questions or discussion points, there may not be ample time for the small group scenarios.
- There is not usually time for more than 3-4 scenarios per small group; we encourage either assigning the scenarios to each group or inviting them to pick 3 -4 among the 7.
- If there are participants with different levels of experience with MI, consider assigning the groups so that they are mixed levels.
- The facilitator should give the groups 3 minutes to role-play and 3 – 5 minutes to review the checklist after the role-play. It is useful to let the groups know they may not have progressed in the scenario as far as they’d like, in the time allotted, but each person should have a chance to play each role (smoker, counselor, observer with checklist).
- Orient learners to the use of the checklist, which is to be used as a guide for feedback after the role-play. The observer can circle or check individual items on the checklist, or write notes for specific items. The goal is to provide an anchor for discussing MI-specific skills that may have been used, or may not have been used in the role-play.
- Encourage the use of the laminated card during the role-play sessions, and encourage participants to keep the card with them to utilize in their real-life clinical encounters.

**Useful MI References**

- Rollnick S, Miller W, Butler C., *Motivational Interviewing in Health Care: Helping Patients Change Behavior,* New York, NY: Guilford Press; 2008.
- <http://www.motivatehealthyhabits.com/index.html>
- <https://www.youtube.com/watch?v=s3MCJZ7OGRk>
- <https://www.kognito.com/changetalk/Change_Talk_AAP_Kognito_Overview.pdf>
