# Supplementary material for: Motivational Interviewing: A High-Yield Interactive Session for Medical Trainees and Professionals to Help Tobacco Users Quit
Source: MedEdPORTAL. 2019 Aug 23;15:10831. doi: 10.15766/mep_2374-8265.10831 (PMC6868517; doi:10.15766/mep_2374-8265.10831)
Supplement: Supplementary file 1 — A. MI Presentation.pptx B. MI Workshop Scenarios.docx C. Checklist for MI.docx D. MI Laminated Card.pptx E. Resident Survey.docx F. MI Facilitator Guide.docx [file mep-15-10831-s001.zip › _Educational_Summary_Report_10831.pdf]

MedEdPORTAL® The AAMC Journal of Teaching and Learning Resources

Original Publication Open Access

Motivational Interviewing: A High-Yield Interactive Session for Medical Trainees and Professionals to Help Tobacco Users Quit

Rachel Boykan, MD*, Julie Gorzkowski, MSW, LSW, Jyothi Marbin, MD, Jonathan Winickoff, MD, MPH

*Corresponding author: rachel.boykan@stonybrookmedicine.edu

Abstract

Introduction: Motivational interviewing (MI) is a collaborative patient-focused counseling technique that is effective in promoting smoking cessation but is not consistently taught/practiced in training. **Methods:** This training session was implemented in a pediatric residency training program and also given four times to pediatric practitioners as part of a 2-day tobacco training sponsored by the American Academy of Pediatrics (AAP). Pediatric residents ($N = 33$) participated in a 1-hour interactive session focused on addressing tobacco. Knowledge was assessed with pre- and 6-month postsurveys. Retention of skills was evaluated between 6 and 9 months posttraining by resident performance on two scenarios with standardized patients, which was scored utilizing the Behavior Change Counseling Index (BECCI), by two MI-trained physicians. AAP trainees ($N = 115$) participated in tobacco trainings with a session dedicated to MI; sessions were evaluated by pre- and posttests. **Results:** Residents who completed the session ($n = 12$) performed significantly better on eight of 10 items of the BECCI and on the overall BECCI score ($p < .001$) compared with those who had not completed the session ($n = 12$). Feedback on AAP training sessions ($N = 115$) indicated that practitioners felt able to perform MI and incorporate MI into practice. The percentage of trainees who felt comfortable counseling about tobacco doubled from pre- to posttraining. **Discussion:** A hands-on MI training session provided pediatric residents and practicing clinicians with knowledge and skills to address tobacco use with patients/families. The session is easily incorporated into different training environments.

Keywords
Motivational Interviewing, Tobacco Smoke Exposure, Tobacco, Pediatrics, Preventive Medicine

Educational Objectives

By the end of this activity, learners will be able to:

1. Define principles and components of motivational interviewing (MI).
2. Describe ways to implement MI techniques to address tobacco use with patients/families.
3. Develop skills through practice to utilize MI techniques to address tobacco use with patients/families.

Introduction

Tobacco smoke exposure (TSE) is a well-recognized danger for children. Children exposed to tobacco smoke have an increased risk for sudden infant death syndrome, lower respiratory tract illness, asthma, and ear infections.[1] Healthy children with TSE are 1.4 times more likely to require an emergency room visit; children with asthma and TSE are 2.2 times more likely to be admitted to the hospital.[2] Thirdhand smoke has been more recently described and is the toxic and carcinogenic residue left after the cigarette has been extinguished. Young children are particularly at risk for morbidity from thirdhand smoke because they may have significant dermal and oral exposure.[3,4]

Pediatricians should address TSE with parents/families at every opportunity, but this is not consistently done. Common reasons given by practitioners include lack of time, discomfort in discussing smoking cessation, perceived disinterest on the part of the smoker, and the perception that discussing smoking and TSE is beyond the scope of the clinical visit.[5-8] Furthermore, the majority of residents are not trained to address TSE with their patients and their patients' families/caregivers.[9,10]

Motivational interviewing (MI) is a collaborative patient-focused technique of counseling in which the individual's own motivation for change (in this case, quitting smoking) is reinforced and

Citation:
Boykan R, Gorzkowski J, Marbin J, Winickoff J. Motivational interviewing: a high-yield interactive session for medical trainees and professionals to help tobacco users quit. *MedEdPORTAL*. 2019;15:10831. https://doi.org/10.15766/mep_2374-8265.10831

Copyright © 2019 Boykan et al. This is an open-access publication distributed under the terms of the Creative Commons Attribution license.

utilized to set a goal and plan. MI skills include reflective listening, eliciting motivation, listening more than speaking, and facilitating change by resolving ambivalence. Several studies have demonstrated the effectiveness of MI techniques in smoking cessation. In one randomized controlled trial of 200 smokers followed for 12 months, the group given MI had 5.2 times higher quit rates than the group receiving traditional antismoking advice.[11] Harris et al.[12] studied 450 college students: Those receiving MI had more quit attempts and smoked fewer cigarettes at the end of the 1-month study period than those in the control group.

Curricula for residents in training should be delivered in an efficient and effective manner, with opportunity to integrate concepts with skill building. In one 3-hour multicomponent resident curriculum, which included a didactic component, videos, and role-plays, pediatric residents improved their understanding of MI techniques and frequency of counseling.[13] A more recent systematic review of nine published MI curricula found that it was feasible to teach MI within residency education, and that experiential learning with feedback was most successful.[14] Opportunities for longitudinal practice, with patient encounters or simulation, enhance concepts learned, as was detailed in an MI course taught over 4 weeks with family medicine residents.[15]

There are several curricula available in *MedEdPORTAL* to teach MI skills of varying intensity and focus. Brogan Hartlieb et al.[16] and Azari et al.[17] present longitudinal curricula intended for internal medicine residents, with 12 hours of curriculum to be completed in three to four sessions. Two curricula utilize standardized patients (SPs) for the education of MI.[18,19] In some, MI is taught for use with a particular population: In one case, the focus is women's health issues in military veterans[20]; in another, the focus is on oral health.[21] Although the principles of MI are easily generalized to any specialty, the examples used in these curricula make them less generalizable to pediatrics. The curriculum published by Nelson et al.[22] introduces MI concepts in the context of addressing TSE in a pediatric clinical setting. Our curriculum is similar to that of Nelson et al. in that we present basic MI concepts, but our didactic portion is shorter, allowing for more hands-on practice with the case scenarios. Furthermore, Nelson and colleagues' presentation goes into more detail about "ask, advise, refer," whereas we focus on delving into more specific components of MI. Nelson et al. also introduce prescribing for nicotine replacement, which we do not touch on. Their curriculum includes cases, with some tips for MI, a format similar to ours; however, our cases break down the components of MI with the checklist; more time in our session is focused on developing and practicing MI skills. In summary, we believe that our pediatric-specific curriculum fills a gap and is well suited to incorporation into a busy resident or clinician schedule, with its high-yield didactic portion and hands-on guided practice of specific MI skills provided by the seven different case scenarios and the accompanying checklist.

Methods

We developed a 1-hour training session to teach MI skills, which was implemented and evaluated in two different environments: a pediatric residency training program and as part of a 2-day training ("Asking the Right Questions: Clinicians and Tobacco Control in the Clinical Setting") sponsored by the American Academy of Pediatrics (AAP) Julius B. Richmond Center of Excellence.

Resident Sessions

Thirty-three pediatric residents (from all 3 years of training) attended a required 1-hour educational session. To accommodate residents' schedules, three possible sessions were offered between August and March.

The educational session ran as follows. First, participants watched a 15-minute presentation (Appendix A), given by a facilitator, which provided background information regarding the need to address TSE in the pediatric clinical environment, followed by an introduction to MI principles and an opportunity for group interactive practice with the facilitator at the end of the presentation (slides 18-23 of the PowerPoint presentation, which reviewed a case). The remainder of the session was devoted to small-group application of MI principles by role-play of typical pediatric scenarios in groups of three people each (Appendix B). The recommended session time line is as follows:

- 15 minutes: PowerPoint presentation (Appendix A).
- 35 minutes: Facilitate role-play in groups of three to four. Choose from seven cases (each person should play the counselor once). Role-play should take 2 to 3 minutes, with 3 to 6 minutes for debriefing and facilitated peer feedback. (Appendices B, C, D).
- 10 minutes: Group discussion focusing on difficult encounters, solutions, and lessons learned.

Scenarios

Scenarios were written by one author (Rachel Boykan) and were designed to cover a range of pediatric situations in which caregivers would encounter a smoker, to provide opportunities to simulate real-life experiences. Scenarios 1 and 2 took place in

Copyright © 2019 Boykan et al. This is an open-access publication distributed under the terms of the Creative Commons Attribution license.

the outpatient office setting; scenarios 3, 4, 6, and 7 took place in the hospital setting; and scenario 5 took place in the emergency department. All but scenario 2 involved parents who smoked, but the children were of varying ages and had varying tobacco-related health problems (asthma, infections, Crohn disease), to give the trainee practice with a broad range of situations. Scenario 2 involved a teenager who smoked, which required a different approach. All scenarios involved only one parent (rather than the entire family), to maintain the workshop groupings of three participants each, with one person playing the smoker, one the counselor, and one the observer (who was given a checklist [Appendix C] to provide feedback regarding the interaction). The scenarios could be adapted to be used by groups of two or four, however, and one could add an additional family member—either a child or another parent, if desired; this would not alter the overall purpose of discussing smoking with a smoker, utilizing principles of MI.

Checklist and Feedback

Although the scenarios were different, the same 11-item checklist was used for each scenario so that trainees could practice applying the same MI principles to different situations. The checklist, also developed by Rachel Boykan, encompassed some of the main points of MI as detailed in the 15-minute didactic portion. The checklist was not meant to be scored but rather to be used as a tool to anchor discussion spearheaded by the observer providing feedback. For example, the observer might notice that the trainee asked open-ended questions but did not resist the righting reflex.

Participants were each given a laminated card to use during their practice scenarios, and to keep, to remind them of the main principles and skills learned that day (Appendix D). Each scenario took approximately 5 to 8 minutes to complete, including feedback and discussion among each group. Participants rotated so that each person played the counselor role at least once. During the small-group scenarios, the facilitator (there were several facilitators at the AAP sessions, which had more participants) was present and available for questions or discussion. At the end of all of the scenarios, the large group reported back regarding challenges participants faced and lessons they wanted to share from their own experiences with the role-play. In this way, the discussions after each scenario and the large-group discussion at the completion of the small-group sessions served as a debriefing and an opportunity for clarification of MI principles and techniques. Through the practice of the scenarios and the discussion following, with the checklist as guide, participants became more adept at recognizing and utilizing the MI principles introduced in the 15-minute didactic portion of the workshop.

Prior to the session and at 4 and 7 months post, residents took a web-based survey (Appendix E) evaluating their knowledge/practice regarding smoking cessation and knowledge regarding MI. Additionally, at 6 to 9 months posttraining, 12 PGY 2 and PGY 3 residents who had received the MI training and 12 PGY 1 interns who had not received the MI training participated in two simulated scenarios using SPs, developed specifically for this curriculum, regarding TSE in a pediatric clinical setting.[23] The simulations were videotaped, watched, and scored by two MI-trained (nonblinded) observers using a validated measure of MI—the Behavior Change Counseling Index (BECCI).[24,25]

AAP Sessions

As mentioned earlier, the same session, with exactly the same format, was incorporated into a 2-day in-person tobacco control training for pediatric practitioners, given by the AAP. The MI content was delivered to AAP learners as part of an AAP-sponsored clinical training program called "Asking the Right Questions: Clinicians and Tobacco Control in the Clinical Setting." This clinical program trained pediatric providers in clinical and community strategies to protect children and families from tobacco use and exposure. The MI curriculum was delivered as part of a session on practical strategies to counsel parents and adolescents about tobacco cessation. MI concepts were tailored to tobacco-related case studies, and learners participated in both didactic presentations and interactive role-plays using case scenarios, as detailed earlier with the resident group. We believe that the goals of this MI session aligned with and complemented the overall goals of the 2-day training, and that the session would be easily adaptable to other audiences, including practicing pediatricians and allied health practitioners.

The AAP learners ($N = 115$) were pediatricians and other pediatric health providers who registered to attend a tobacco control training program at the AAP. Approximately 60% were pediatricians, 19% were nurses or nurse practitioners, 11% were allied health professionals, and 10% were "other" (this category included medical assistants, social workers, and office staff). Learners attended the training session in pairs from each practice—each pair consisted of a pediatrician and another person from the same practice who could support implementation of health system changes for tobacco control. Learners came from a variety of practice settings, including

Copyright © 2019 Boykan et al. This is an open-access publication distributed under the terms of the Creative Commons Attribution license.

pediatric group practices, primary care clinics, clinics affiliated with academic/teaching hospitals, and federally qualified health centers. Learners represented 33 US states and two Canadian provinces.

Sessions given at the AAP trainings were evaluated by pre- and posttraining surveys given immediately prior to and following the sessions. No delayed surveys or SP cases were done by these participants.

Results

Resident Group

Of the resident group, all 33 residents completed the initial survey; 26 (79%) and 22 (67%) of the residents completed surveys at 4 and 7 months post, respectively. On all surveys, addressing TSE was important to all residents. In addition, 100% of the residents believed that it was their responsibility to address their patients' smoking. The majority of residents (92% initially and 85% and 100% on subsequent surveys) believed that they should address patients' family members' smoking, and all residents reported addressing TSE by assisting patients/patients' families all or some of the time. Lack of time was the most frequent barrier cited. Resident knowledge regarding counseling for smoking cessation and components of MI improved following intervention. On faculty evaluation of the simulation session, residents who received the MI training performed significantly better on eight of 10 BECCI questions and on their total BECCI score when compared with those who had not received the training (Table 1). Residents who received the training spoke for less time than those who had not received the training (Table 2).

AAP Group

All 115 participants in the AAP trainings completed pre- and posttest surveys. Participants rated their satisfaction with the session and their ability to perform the skills learned on a Likert scale. After the session, 110 participants (96%) agreed or strongly agreed that they felt able to perform MI in practice, 109 (95%) agreed or strongly agreed that the MI training would impact their clinical practice, and 112 (97%) agreed or strongly agreed that the session was a good educational experience. All participants also reported on their comfort level in counseling patients and parents about tobacco use and exposure, pre- and posttraining. Before the training, 43% of the participants reported being comfortable counseling pediatric patients about tobacco, and 45% of the participants reported being comfortable counseling parents of pediatric patients about tobacco. After the training, which included the MI session, 95% of the participants reported being comfortable counseling both pediatric patients and their parents about tobacco ($p < .001$).

Table 1. Comparative Mean Scores on Behavior Change Counseling Index Questions: PGY 1 Versus PGY 2 and PGY 3

Question	PGY	*N*	*M*	*SD*	*SEM*	*p*
1	1	11	1.23	1.08	.326	.445
	2 & 3	10	1.65	1.40	.441	
2	1	12	3.42	0.97	.281	.067
	2 & 3	12	2.54	1.23	.356	
3	1	12	2.12	1.03	.296	<.001
	2 & 3	12	3.71	0.62	.179	
4	1	12	1.71	1.12	.323	<.001
	2 & 3	12	3.33	0.62	.177	
5	1	12	1.58	1.24	.358	<.001
	2 & 3	12	3.46	0.75	.217	
6	1	12	2.46	0.94	.272	.002
	2 & 3	12	3.54	0.54	.156	
7	1	12	0.83	0.94	.271	.001
	2 & 3	12	2.34	1.09	.315	
8	1	12	1.79	0.81	.234	<.001
	2 & 3	12	3.21	0.69	.199	
9	1	12	1.92	0.79	.229	<.001
	2 & 3	12	3.25	0.69	.199	
10	1	12	1.79	0.84	.242	<.001
	2 & 3	12	3.67	0.62	.177	
Total score	1	12	1.87	0.71	.204	<.001
	2 & 3	12	3.08	0.57	.165	

Abbreviation: *SEM*, standard error of the mean.

Discussion

A brief hands-on MI training session provided pediatric residents and practicing pediatric clinicians with knowledge and skills to address tobacco use in their patients/patients' families, filling an important gap in pediatric residency training. Based on resident pre- and postsurveys, residents gained knowledge regarding MI concepts. Residents who participated in the curriculum performed significantly better on the simulated cases 6 to 9 months after the session (sustained improvement), indicating that skills learned during the brief session were maintained over time.

Such results are encouraging but should be taken in the context of several limitations. We did not conduct baseline assessments regarding participants' experience in practicing MI, so postcurricular improvement in the resident group (those who had the curriculum prior to the simulation were second- and third-year residents) could be explained by

Table 2. Cross Tabulation of Group by Time Spoken ($p = .001$)

	Spoke for More Than Half the Time		Spoke for Half the Time or Less		Total	
Group	*n*	%	*n*	%	*N*	%
PGY 1	10	83.3	2	16.7	12	100
PGY 2 & PGY 3	2	16.7	10	83.3	12	100
Total	12	50.0	12	50.0	24	100

Copyright © 2019 Boykan et al. This is an open-access publication distributed under the terms of the Creative Commons Attribution license.

improvement in skills naturally gained throughout the course of residency. Similarly, we did not assess baseline knowledge or experience regarding MI with the AAP group, which was a diverse mix of pediatricians and other pediatric health care providers. The two faculty members who scored the simulation sessions were not blinded to participant group assignments, potentially biasing their evaluations. In two cases, interns who had not participated in the MI session scored higher than their peers who had not participated; however, on further questioning, they had received MI training prior to starting residency. Although we believe that the session was easily adapted to different learners (residents and seasoned practitioners at different levels), we recognize that with the relatively small number of learners and assessments, our results may not be generalizable to others. We saw sustained improvement in skills over time, as evidenced by our residents' performance on the BECCI; however, it would be important for others utilizing these materials to conduct their own assessments, as their results could vary from ours.

The preceding limitations aside, this training session has several strengths. First, MI concepts are presented in an easy-to-learn and focused, relevant pediatric context—that of addressing tobacco smoking among parents and patients. This is a topic that pediatric trainees and practitioners are often not comfortable addressing; skills learned in this interactive hour are highly applicable. Second, although the principles of MI are addressed in other curricula, putting these skills into practice through role-play, with guided feedback, fills a gap not provided by other existing curricula.

The session requires few resources and little setup: a screen to view the PowerPoint presentation and a room large enough to accommodate groups of three for the given number of participants. The laminated cards were considered useful by the participants but are not necessary for the session itself. The facilitator should have knowledge of MI and should be able to distinguish MI concepts in dialogue to guide participants through the role-play, when necessary. Advanced training in MI should not be necessary for the purposes of this curriculum; however, interested individuals could utilize some of the training resources listed in the Facilitator Guide (Appendix F) to hone skills prior to leading the session, if desired.

The sessions given at the AAP were the same as those given to the resident group. The 15-minute didactic portion was familiar to some learners and served as a review, whereas for others it was new information. The difference in learner levels was evident to facilitators during the role-play portion, as some participants were better able to utilize the principles easily, whereas others struggled. We did not divide the groups according to level of expertise, but it would be helpful to arrange the groups so that learners without prior training in MI are mixed with those with more experience. We did find, however, that some individuals who considered themselves to be more expert in MI techniques still received valuable feedback from their peers and believed that the session was useful for honing their own skills.

The session for residents was held three times to ensure that all residents attended at least once. As a result, some residents participated more than once in the session. We did not evaluate whether subsequent participation served as a booster session for these residents, although it would be a reasonable conclusion. With subsequent evaluations of this curriculum, we might evaluate a dose-response effect of booster sessions.

Certainly, one cannot expect mastery of MI in a 1-hour session. However, given the significant time constraints inherent in resident and postresidency training, we believe that the 1-hour session presents learning in a high-yield and practical manner. Similarly, although we did not give this session in an office setting, we imagine that pediatricians and other health care practitioners could utilize a 1-hour training on-site. Given the success of the session with the wide range of AAP participants, we believe that the session would be easily generalizable to other convenient settings for clinicians. Regardless of the setting and learner level, participants should be prepared to continue to practice these skills in their clinical encounters.

The specific focus on MI to address tobacco use and exposure may be useful in a broad range of contexts. In addition to residents and other pediatric practitioners, it could be used for medical or nursing student training and incorporated into the clinical rotations, in which students would have a chance to practice these skills in the clinical setting.

We continue to use this MI training session for each incoming class of pediatric residents at our institution. Future directions include continued pre- and postassessments of learners, by both survey and simulation,[23] for longitudinal assessment of skills. It would be informative to see if repetition of the session for residents during each year of training reinforces MI skills. Finally, the simulation session,[23] initially utilized for the evaluation of the effectiveness of this session, may serve as an opportunity to reinforce MI skills learned—we encourage those utilizing this resource to consider using that one as well.

Copyright © 2019 Boykan et al. This is an open-access publication distributed under the terms of the Creative Commons Attribution license.

Appendices

A. MI Presentation.pptx

B. MI Workshop Scenarios.docx

C. Checklist for MI.docx

D. MI Laminated Card.pptx

E. Resident Survey.docx

F. MI Facilitator Guide.docx

All appendices are peer reviewed as integral parts of the Original Publication.

Rachel Boykan, MD: Associate Professor, Department of Pediatrics, Renaissance School of Medicine at Stony Brook University

Julie Gorzkowski, MSW, LSW: Director, Adolescent Health Promotion, American Academy of Pediatrics

Jyothi Marbin, MD: Associate Professor, Department of Pediatrics, University of California, San Francisco, School of Medicine

Jonathan Winickoff, MD, MPH: Professor, Pediatrics, Harvard Medical School; Professor, Pediatrics, MassGeneral Hospital for Children; Director of Translational Research, American Academy of Pediatrics Julius B. Richmond Center of Excellence; Director of Pediatric Research, Tobacco Research and Treatment Center, Massachusetts General Hospital

Acknowledgments

We would like to thank Amy Whittle, MD, for her input in the development of the workshop.

Disclosures

None to report.

Funding/Support

None to report.

Prior Presentations

Boykan R, Blair R, Messina CR. Motivational interviewing to improve quitline referrals—a resident education initiative. Poster presented at: AAP National Conference & Exhibition, Section on Tobacco Control; September 15, 2017; Chicago, IL.

Ethical Approval

Reported as not applicable.

References

1. *The Health Consequences of Smoking—50 Years of Progress: A Report of the Surgeon General*. Atlanta, GA: U.S. Department of Health and Human Services, Centers for Disease Control and Prevention, National Center for Chronic Disease Prevention and Health Promotion, Office on Smoking and Health; 2014.
2. Jin Y, Seiber EE, Ferketich AK. Secondhand smoke and asthma: what are the effects on healthcare utilization among children? *Prev Med.* 2013;57(2):125-128. https://doi.org/10.1016/j.ypmed.2013.05.003
3. Matt GE, Quintana PJE, Destaillats H, et al. Thirdhand tobacco smoke: emerging evidence and arguments for a multidisciplinary research agenda. *Environ Health Perspect.* 2011;119(9):1218-1226. https://doi.org/10.1289/ehp.1103500
4. Section on Tobacco Control. Clinical practice policy to protect children from tobacco, nicotine, and tobacco smoke. *Pediatrics*. 2015;136(5):1008-1017. https://doi.org/10.1542/peds.2015-3108
5. Winickoff JP, McMillen RC, Carroll BC, et al. Addressing parental smoking in pediatrics and family practice: a national survey of parents. *Pediatrics*. 2003;112(5):1146-1151. https://doi.org/10.1542/peds.112.5.1146
6. Tanski SE, Klein JD, Winickoff JP, Auinger P, Weitzman M. Tobacco counseling at well-child and tobacco-influenced illness visits: opportunities for improvement. *Pediatrics*. 2003;111(2):e162-e167. https://doi.org/10.1542/peds.111.2.e162
7. Cabana MD, Rand C, Slish K, Nan B, Davis MM, Clark N. Pediatrician self-efficacy for counseling parents of asthmatic children to quit smoking. *Pediatrics*. 2004;113(1):78-81. https://doi.org/10.1542/peds.113.1.78
8. Burnett KF, Young PC. Ask, advise, assist: pediatricians and passive smoke exposure. *Clin Pediatr (Phila)*. 1999;38(6):339-345. https://doi.org/10.1177/000992289903800604
9. Hymowitz N, Schwab JV. Pediatric Residency Training Director Tobacco Survey II. *Pediatrics*. 2012;130(4):712-716. https://doi.org/10.1542/peds.2011-3570
10. Dodge RAB, Cabana MD, O'Riordan MA, Heneghan A. What factors are important for pediatric residents' smoking cessation counseling of parents? *Clin Pediatr (Phila)*. 2008;47(3):237-243. https://doi.org/10.1177/0009922807308182
11. Soria R, Legido A, Escolano C, Lopez Yeste A, Montoya J. A randomised controlled trial of motivational interviewing for smoking cessation. *Br J Gen Pract*. 2006;56(531):768-774.
12. Harris KJ, Catley D, Good GE, Cronk NJ, Harrar S, Williams KB. Motivational interviewing for smoking cessation in college students: a group randomized controlled trial. *Prev Med*. 2010;51(5):387-393. https://doi.org/10.1016/j.ypmed.2010.08.018
13. Scal P, Hennrikus D, Ehrlich L, Ireland M, Borowsky I. Preparing residents to counsel about smoking. *Clin Pediatr (Phila)*. 2004;43(8):703-708. https://doi.org/10.1177/000992280404300803
14. Dunhill D, Schmidt S, Klein R. Motivational interviewing interventions in graduate medical education: a systematic review of the evidence. *J Grad Med Educ*. 2014;6(2):222-236. https://doi.org/10.4300/JGME-D-13-00124.1
15. Kaltman S, WinklerPrins V, Serrano A, Talisman N. Enhancing motivational interviewing training in a family medicine clerkship. *Teach Learn Med*. 2015;27(1):80-84. https://doi.org/10.1080/10401334.2014.979179

Copyright © 2019 Boykan et al. This is an open-access publication distributed under the terms of the Creative Commons Attribution license.

16. Brogan Hartlieb K, Engle B, Obeso V, Pedoussaut MA, Merlo LJ, Brown DR. Advanced patient-centered communication for health behavior change: motivational interviewing workshops for medical learners. *MedEdPORTAL*. 2016;12:10455. https://doi.org/10.15766/mep_2374-8265.10455

17. Azari S, Ratanawongsa N, Hettema J, et al. A skills-based curriculum for teaching motivational interviewing-enhanced screening, brief intervention, and referral to treatment (SBIRT) to medical residents. *MedEdPORTAL*. 2015;11:10080. https://doi.org/10.15766/mep_2374-8265.10080

18. Brown D, Mechaber A, Trapido E, et al. Tobacco cessation objective structured clinical examination (OSCE). *MedEdPORTAL*. 2009;5:5102. https://doi.org/10.15766/mep_2374-8265.5102

19. Kolasa K, Craven K, Byrd J, Merricks P. Helping your patient change: a patient-centered behavioral counseling presentation for second-year medical students. *MedEdPORTAL*. 2015;11:10166. https://doi.org/10.15766/mep_2374-8265.10166

20. Day H, Scott R, Fulmer V, et al. Women's health issues in military veterans: standardized patient cases in motivational interviewing, a case materials guide. *MedEdPORTAL*. 2013;9:9539. https://doi.org/10.15766/mep_2374-8265.9539

21. Romito L, Coan L, Christen A. Practical counseling and communication strategies: tobacco cessation. *MedEdPORTAL*. 2013;9:9324. https://doi.org/10.15766/mep_2374-8265.9324

22. Nelson K, Goodwin K, State R, Hobson-Rohrer W. Child tobacco smoke exposure: interventions for busy physicians. *MedEdPORTAL*. 2016;12:10344. https://doi.org/10.15766/mep_2374-8265.10344

23. Boykan R, Blair R, Baldelli P, Owens S. Using motivational interviewing to address tobacco cessation: two standardized patient cases for pediatric residents. *MedEdPORTAL*. 2019;15:10807. https://doi.org/10.15766/mep_2374-8265.10807

24. Lane C, Huws-Thomas M, Hood K, Rollnick S, Edwards K, Robling M. Measuring adaptations of motivational interviewing: the development and validation of the Behavior Change Counseling Index (BECCI). *Patient Educ Couns*. 2005;56(2):166-173. https://doi.org/10.1016/j.pec.2004.01.003

25. The Behaviour Change Counselling Index (BECCI): manual for coding behaviour change counselling. Motivational Interviewing Network of Trainers website. https://motivationalinterviewing.org/sites/default/files/BECCIManual.pdf. Published 2002. Accessed January 15, 2019.

Received: January 16, 2019
Accepted: April 29, 2019
Published: August 23, 2019

Copyright © 2019 Boykan et al. This is an open-access publication distributed under the terms of the Creative Commons Attribution license.
